# Supplementary material for: The first nationwide study on facing and solving ethical dilemmas among healthcare professionals in Slovenia
Source: PLoS One. 2020 Jul 14;15(7):e0235509. doi: 10.1371/journal.pone.0235509 (PMC7360038; doi:10.1371/journal.pone.0235509)
Supplement: S1 Appendix — (DOCX) [file pone.0235509.s001.docx]

**APPENDIX 1: Questionnaire for healthcare professionals**

**A SURVEY ON HEALTHCARE PROFESSIONALS’ ETHICAL DILEMMAS**

Dear healthcare worker,

This is a questionnaire accompanying the study entitled “*The first nationwide study on facing and solving ethical di-lemmas among healthcare professionals in Slovenia”.*

Ethics is the science of morality. Medical ethics is concerned with applying ethical and moral principles in day-to-day challenges of the healthcare professional. Ethical dilemmas arise when the healthcare professional is faced with a choice between two or more opposing moral dilemmas.

We are aware that you yourself might have already confronted such dilemmas. We are therefore interested in learning more about your ability to recognize and resolve these situations in an ethically appropriate manner. Especially, whether you know where to seek support, whether you are satisfied with the way ethical dilemmas are currently being addressed in your hospital, and what your expectations are with respect to how such a system should operate. Medical ethics committees are recognized world-wide as a well-established means for resolving ethical dilemmas. With this survey, we intend to explore their role in Slovenian healthcare institutions. The study was approved by the National Medical Ethics Committee of the Republic of Slovenia.

We hope to see an enthusiastic response from the healthcare community. You can greatly help us in this by filling out this questionnaire; your participation in the study is entirely anonymous. The results will be made publicly available.

We thank you in advance for taking part in the study.

Study authors:

Full Prof. Dr. Štefan Grosek, MD,

[stefan.grosek@kclj.si](mailto:stefan.grosek@kclj.si); +386 41 684 206

Assoc. Prof. Dr. Blaž Ivanc, LLB, legal expert,

[blaz.ivanc@zf.uni-lj.si](mailto:blaz.ivanc@zf.uni-lj.si); +386 13 00 11 69

Assist. Prof. Dr. Urh Grošelj, MD, MA, teaching assistant,

Miha Oražem, MD,

Jon Grošelj, BA, economist, philosopher, and sociologist

Rok Kučan, medical student,

Vanja Ida Erčulj, PhD, statistician, and psychologist

Jaro Lajovic, MD.

*The questionnaire is protected by copyright law. Copying is prohibited. Reuse is allowed only with prior written approval by the study authors.*

1. **GENERAL INFORMATION**
   1. GENDER

- male
- female
  1. AGE

Please fill in: _______ years

- 1. RELIGIOUS/WORLD VIEWS

*Religions are listed in descending order according to the number of adherents from the 2002 census.*

- Catholic
- Muslim
- Orthodox
- Protestant
- atheist
- undeclared
- I decline to answer
- other (please specify): ______________________________
  1. TYPE OF INSTITUTION
- secondary level institution
- tertiary level institution
- other (please specify): ______________________________
  1. EMPLOYMENT
- healthcare assistant
- nurse
- nursing graduate
- physiotherapist
- work therapist
- lab manager
- radiologist
- clinical psychologist
- medical trainee:
  - - please specify area of specialization: ______________________________

____________________________________________________________

- - - other (please specify): __________________________________________

____________________________________________________________

- 1. IN WHICH HOSPITAL UNIT DO YOU SPEND MOST OF YOUR WORK TIME?

*Please choose only one answer.*

- reception clinic
- clinic
- emergency wards
- hospital wards
- intensive care units
- diagnostic wards
- surgical wards
- elsewhere (please specify): ______________________________
  1. HOW LONG HAVE YOU BEEN EMPLOYED AT YOUR CURRENT HEALTHCARE INSTITUTION?

Please fill in: _______ years

- 1. WHAT IS YOUR TOTAL EMPLOYMENT PERIOD?

Please fill in: _______ years

1. **MANAGING AND RESOLVING ETHICAL DILEMMAS**

*This section includes questions pertaining to ethical dilemmas in your day-to-day work and interpersonal relations in the workplace. Ethical dilemmas are listed in part 2.2.*

- 1. HOW OFTEN ARE YOU FACED WITH ETHICAL DILEMMAS?
- very rarely
- rarely
- not frequently, not rarely
- frequently
- very frequently
  1. PLEASE ESTIMATE HOW OFTEN YOU ENCOUNTER ETHICAL DILEMMAS IN THE DOMAINS LISTED BELOW IN YOUR PROFESSIONAL WORK. *Please indicate by putting a cross in the appropriate box.*

|  | very rarely | rarely | not frequently, not rarely | frequently | Very frequently |
| --- | --- | --- | --- | --- | --- |
| new modes of treatment and diagnostic procedures |  |  |  |  |  |
| organ transplantation |  |  |  |  |  |
| end-of-life treatment withdrawal |  |  |  |  |  |
| suboptimal working conditions due to poor interpersonal relations on the ward |  |  |  |  |  |
| disagreement with an individual’s professional work |  |  |  |  |  |
| lack of response to adverse events in patient management |  |  |  |  |  |
| learning on patients |  |  |  |  |  |
| social inequality or withdrawal of basic healthcare insurance |  |  |  |  |  |
| biomedical research |  |  |  |  |  |
| a patient’s consent to undergo a diagnostic or therapeutic procedure |  |  |  |  |  |
| allocation of limited resources |  |  |  |  |  |
| recognizing patient’s best interest |  |  |  |  |  |
| preserving patient’s dignity |  |  |  |  |  |
| protection of patient information |  |  |  |  |  |
| relations between HCPs and patients (or their relatives) |  |  |  |  |  |
| involuntary hospitalization |  |  |  |  |  |
| patient’s right to refuse treatment |  |  |  |  |  |
| refusal of vaccines |  |  |  |  |  |
| insufficient availability of palliative care |  |  |  |  |  |
| waiting periods for diagnostics or therapeutic treatment |  |  |  |  |  |
| other (please specify): |  |  |  |  |  |

- 1. HOW DO YOU PROCEED WHEN FACED WITH AN ETHICAL DILEMMA? *It is possible to choose more than one answer.*
- I discuss it with the head of the department.
- I discuss it with co-workers.
- I call a medical council meeting.
- I consult the hospital ethics committee.
- I consult the national medical ethics committee (the National Medical Ethics Committee of the Republic of Slovenia)
- I consult the Committee for Legal and Ethical Issues of the Medical Chamber of Slovenia.
- I consult the patient’s legal representative.
- I consult the Human Rights Ombudsman.
- I consult the hospital chaplain.
- I try to resolve the issue with mediation.
- I try to resolve the issue by contacting the media.
- I try to resolve the issue in family circles.
- I decide on my own.
- Other (please specify): ______________________________
  1. DOES YOUR HOSPITAL MAINTAIN A STANDARD PROCEDURE FOR RESOLVING ETHICAL DILEMMAS?
- yes
- I don’t know
- no
  1. CAN YOU RECALL A CASE FROM YOUR MEDICAL PRACTICE WHICH WOULD REQUIRE ETHICAL JUDGMENT BUT IT HAS IN FACT NOT RECEIVED IT?
- yes
- I don’t know
- no
  1. PLEASE INDICATE WHAT MODE OF INSTRUCTION ON ETHICS AND MORALITY IN HEALTHCARE IS IN YOUR OPINION MOST IMPORTANT?
- self-study
- learning from more experienced peers
- family
- educational system or university
- workshops
- hands-on approach, learning through practice
- other (please specify): ______________________________
  1. TO WHAT EXTENT DO YOU AGREE WITH THE FOLLOWING STATEMENT? *“The hospital staff needs regular formal trainings in healthcare ethics.”*
- I strongly disagree
- I disagree
- I neither agree neither disagree
- I agree
- I strongly agree

1. **MEDICAL ETHICS COMMITTEE IN YOUR HEALTHCARE INSTITUTION**
   1. IN YOUR OPINION, HOW IMPORTANT IS THE ROLE OF THE MEDICAL ETHICS COMMITTEE IN RESOLVING ETHICAL DILEMMAS?

- not important at all
- not important
- neither important neither unimportant
- important
- very important
  1. PLEASE CHOOSE FROM THE LIST BELOW THREE MOST IMPORTANT AREAS OF RESPONSIBILITY OF THE MEDICAL ETHICS COMMITTEE.
- review of difficult cases
- ethical consultation for employees (in session format)
- ethical consultation in the ward (with patients)
- education of healthcare staff
- improving communication
- conflict resolution
- preparation of guidelines or protocols
- support of patients in giving them a stronger voice in decision-making
- legal protection of physicians in decision-making process
- moral support for healthcare professionals
- allocation of limited resources
- counseling hospital management staff
- improving the quality of healthcare
- assessment upon introduction of novel treatment methods
- other (please specify): ______________________________
  1. DOES THE HEALTHCARE INSTITUTION YOU ARE EMPLOYED WITH HAVE ITS OWN MEDICAL ETHICS COMMITTEE?
- yes
- I don’t know
- no
  1. DOES THE HEALTHCARE INSTITUTION YOU ARE EMPLOYED WITH OFFER YOU THE POSSIBILITY TO HAVE ETHICAL CONSULTATIONS WITH THE MEDICAL ETHICS COMMITTEE?
- yes
- I don’t know
- no
  1. HOW MANY TIMES DID YOU CONSULT THE MEDICAL ETHICS COMMITTEE OF THE HEALTHCARE INSTITUTION YOU ARE EMPLOYED WITH IN 2014?

Please fill in: _______ times
