# Supplementary material for: The first nationwide study on facing and solving ethical dilemmas among healthcare professionals in Slovenia
Source: PLoS One. 2020 Jul 14;15(7):e0235509. doi: 10.1371/journal.pone.0235509 (PMC7360038; doi:10.1371/journal.pone.0235509)
Supplement: S1 File — (DOCX) [file pone.0235509.s002.docx]

The seven-step approach to questionnaire development, as recommended by the AMEE guidelines, was followed:

Step 1: Literature review

An extensive literature review was performed to formulate a clear definition of the construct under consideration. Existing measurement instruments were reviewed and adapted to the Slovene context.

Step 2: Interviews / focus groups

Interviews with some members of ethics committees in the tertiary level university hospital were conducted in this phase to obtain a deeper understanding of the research area and ethical dilemmas that are reported more frequently by medical staff.

Step 3: Synthesise the literature review and interviews

In this phase, a comprehensive list of indicators for the construct was made by merging the results of the literature review and interviews/focus groups.

Step 4: Develop items

A larger number of items (n = 33) with specific ethical dilemmas were included in the questionnaire based on expert judgement and the literature review. Some of these items were dropped on the basis of the results of the pilot testing. Unambiguous items were developed to avoid negatively worded items and biased language. The vocabulary of the target population was used.

Step 5: Expert validation:

Different experts (physicians, nurses, members of Hospital and National Ethics Committees, lawyers, and laypersons) evaluated the content of the questionnaire. The questionnaire was changed accordingly. It was also checked for wording and possible double-barrelled, confusing, and misleading questions by two psychometricians.

Step 6: Cognitive interviews

The questionnaire was reviewed by a smaller number of potential respondents (physicians, nurses, and other HCPs). The meaning of each question was thoroughly discussed.

Step 7: Pilot testing:

The pilot questionnaire was answered by 35 healthcare professionals with different occupational profiles. Participants commented on the questionnaire and pointed out possible difficulties they had in understanding or answering certain questions.  On the basis of comments and item variance, some items were omitted from the final questionnaire. The validity and reliability of the questionnaire were examined as part of the main research phase after collecting data with the revised questionnaire (and not as part of pilot testing as proposed in the guidelines). Exploratory factor analysis on the whole Slovenian sample showed that all the items measuring the frequency of ethical dilemmas loaded substantially (loadings > 0.50) on the same factor. The validity of the questionnaire was assessed further by investigating correlations between a general question on the frequency of ethical dilemmas and questions on the frequency of each specific dilemma. The correlation coefficients varied between 0.27 and 0.52 and were all statistically significant (p < 0.001). The reliability of the questionnaire was also examined. The questionnaire exhibited high internal consistency (Cronbach α = 0.93). Test-retest reliability was not assessed to ensure complete respondent anonymity. The latter was crucial for the validity of the obtained results.
