# Supplementary material for: The first nationwide study on facing and solving ethical dilemmas among healthcare professionals in Slovenia
Source: PLoS One. 2020 Jul 14;15(7):e0235509. doi: 10.1371/journal.pone.0235509 (PMC7360038; doi:10.1371/journal.pone.0235509)
Supplement: S1 Table — (DOCX) [file pone.0235509.s003.docx]

**S1_Table 1: Association between the type of institution and physicians’ reactions when faced with ethical dilemmas (results of univariate logistic regression with tertiary level institutions as the reference category)**

|  | Secondary level institution (n = 25) | | Tertiary level institution (n = 51) | |  |  |
| --- | --- | --- | --- | --- | --- | --- |
|  | no | yes | no | yes | OR (95% CI) | P-value |
| Discuss with head of department | 8 (32) | 17 (68) | 11 (21.6) | 40 (78.4) | 0.6 (0.2; 1.7) | 0.326 |
| Discuss with colleagues | 3 (12) | 22 (88) | 4 (7.8) | 47 (92.2) | 0.6 (0.1; 3) | 0.559 |
| Convene a medical council meeting | 17 (68) | 8 (32) | 23 (45.1) | 28 (54.9) | 0.4 (0.1; 1.1) | 0.064 |
| Discuss with hospital medical ethics committee | 24 (96) | 1 (4) | 33 (64.7) | 18 (35.3) | 0.1 (0.01; 0.6) | **0.015** |
| Discuss with national medical ethics committee (Republic of Slovenia National Medical Ethics Committee) | 23 (92) | 2 (8) | 49 (96.1) | 2 (3.9) | 2.1 (0.3; 16.1) | 0.463 |
| Discuss with Legal-ethical committee of the Medical Chamber of Slovenia | 25 (100) | 0 (0) | 51 (100) | 0 (0) |  |  |
| Discuss with Patient Rights Advocate | 25 (100) | 0 (0) | 48 (94.1) | 3 (5.9) |  |  |
| Discuss with Human Rights Ombudsman | 25 (100) | 0 (0) | 49 (96.1) | 2 (3.9) |  |  |
| Consult with hospital chaplain | 24 (96) | 1 (4) | 51 (100) | 0 (0) |  |  |
| Resolve dilemma through mediation | 22 (88) | 3 (12) | 51 (100) | 0 (0) |  |  |
| Contact the media | 25 (100) | 0 (0) | 51 (100) | 0 (0) |  |  |
| Discuss within my family circle | 19 (76) | 6 (24) | 48 (94.1) | 3 (5.9) | 5.1 (1.1; 22.3) | **0.032** |
| Decide alone | 18 (72) | 7 (28) | 45 (88.2) | 6 (11.8) | 2.9 (0.9; 9.9) | 0.085 |

* OR = odds ratio adjusted for hospital; CI = confidence interval
