# Supplementary material for: The first nationwide study on facing and solving ethical dilemmas among healthcare professionals in Slovenia
Source: PLoS One. 2020 Jul 14;15(7):e0235509. doi: 10.1371/journal.pone.0235509 (PMC7360038; doi:10.1371/journal.pone.0235509)
Supplement: S2 Table — (DOCX) [file pone.0235509.s004.docx]

**S2_Table 2: Association between the type of institution and nurses’ reactions when faced with ethical dilemmas (results of univariate logistic regression with tertiary level institutions as the reference category)**

|  | Secondary level institution (n = 122) | | Tertiary level institution (n = 198) | |  |  |
| --- | --- | --- | --- | --- | --- | --- |
|  | no | yes | no | yes | OR (95% CI) | P-value |
| Discuss with head of department | 48 (39.3) | 74 (60.7) | 116 (58.6) | 82 (41.4) | 2.2 (1.4; 3.5) | **0.001** |
| Discuss with colleagues | 5 (4.1) | 117 (95.9) | 7 (3.5) | 191 (96.5) | 0.9 (0.3; 2.8) | 0.797 |
| Convene a medical council meeting | 119 (97.5) | 3 (2.5) | 194 (98) | 4 (2) | 1.2 (0.3; 5.6) | 0.795 |
| Discuss with hospital medical ethics committee | 120 (98.4) | 2 (1.6) | 190 (96) | 8 (4) | 0.4 (0.1; 1.9) | 0.246 |
| Discuss with national medical ethics committee (Republic of Slovenia National Medical Ethics Committee) | 122 (100) | 0 (0) | 198 (100) | 0 (0) |  |  |
| Discuss with Legal-ethical committee of the Medical Chamber of Slovenia | 122 (100) | 0 (0) | 197 (99.5) | 1 (0.5) |  |  |
| Discuss with Patient Rights Advocate | 116 (95.9) | 5 (4.1) | 194 (98) | 4 (2) | 2.1 (0.6; 7.9) | 0.279 |
| Discuss with Human Rights Ombudsman | 122 (100) | 0 (0) | 197 (99.5) | 1 (0.5) |  |  |
| Consult with hospital chaplain | 122 (100) | 0 (0) | 193 (97.5) | 5 (2.5) |  |  |
| Resolve dilemma through mediation | 115 (94.3) | 7 (5.7) | 195 (98.5) | 3 (1.5) | 4 (1; 15.6) | **0.049** |
| Contact the media | 122 (100) | 0 (0) | 198 (100) | 0 (0) |  |  |
| Discuss within my family circle | 117 (95.9) | 5 (4.1) | 192 (97) | 6 (3) | 1.4 (0.4; 4.6) | 0.612 |
| Decide alone | 115 (94.3) | 7 (5.7) | 181 (91.4) | 17 (8.6) | 0.6 (0.3; 1.6) | 0.351 |

* OR = odds ratio adjusted for hospital; CI = confidence interval
