# Supplementary material for: The first nationwide study on facing and solving ethical dilemmas among healthcare professionals in Slovenia
Source: PLoS One. 2020 Jul 14;15(7):e0235509. doi: 10.1371/journal.pone.0235509 (PMC7360038; doi:10.1371/journal.pone.0235509)
Supplement: S3 Table — (DOCX) [file pone.0235509.s005.docx]

**S3_Table 3: Association between the type of institution and other HCPs’ reactions when faced with ethical dilemmas (results of univariate logistic regression with tertiary level institutions as the reference category)**

|  | Secondary level institution (n = 29) | | Tertiary level institution  (n = 57) | |  |  |
| --- | --- | --- | --- | --- | --- | --- |
|  | no | yes | no | yes | OR (95% CI) | P-value |
| Discuss with head of department | 6 (20.7) | 23 (79.3) | 27 (47.4) | 30 (52.6) | 3.4 (1.2; 9.7) | **0.019** |
| Discuss with colleagues | 2 (6.9) | 27 (93.1) | 7 (12.3) | 50 (87.7) | 1.9 (0.4; 9.7) | 0.447 |
| Convene a medical council meeting | 28 (96.6) | 1 (3.4) | 57 (100) | 0 (0) |  |  |
| Discuss with hospital medical ethics committee | 29 (100) | 0 (0) | 55 (96.5) | 2 (3.5) |  |  |
| Discuss with national medical ethics committee (Republic of Slovenia National Medical Ethics Committee) | 29 (100) | 0 (0) | 57 (100) | 0 (0) |  |  |
| Discuss with Legal-ethical committee of the Medical Chamber of Slovenia | 29 (100) | 0 (0) | 56 (98.2) | 1 (1.8) |  |  |
| Discuss with Patient Rights Advocate | 29 (100) | 0 (0) | 56 (98.2) | 1 (1.8) |  |  |
| Discuss with Human Rights Ombudsman | 29 (100) | 0 (0) | 56 (98.2) | 1 (1.8) |  |  |
| Consult with hospital chaplain | 29 (100) | 0 (0) | 57 (100) | 0 (0) |  |  |
| Resolve dilemma through mediation | 29 (100) | 0 (0) | 57 (100) | 0 (0) |  |  |
| Contact the media | 29 (100) | 0 (0) | 56 (98.2) | 1 (1.8) |  |  |
| Discuss within my family circle | 27 (93.1) | 2 (6.9) | 55 (96.5) | 2 (3.5) | 2 (0.3; 15.3) | 0.489 |
| Decide alone | 18 (62.1) | 11 (37.9) | 54 (94.7) | 3 (5.3) | 11 (2.8; 43.9) | **0.001** |

* OR = odds ratio adjusted for hospital; CI = confidence interval
