# Supplementary material for: The first nationwide study on facing and solving ethical dilemmas among healthcare professionals in Slovenia
Source: PLoS One. 2020 Jul 14;15(7):e0235509. doi: 10.1371/journal.pone.0235509 (PMC7360038; doi:10.1371/journal.pone.0235509)
Supplement: Attachment — Submitted filename: PONE-D-20-08547_reviewer 05042020.pdf [file pone.0235509.s007.pdf]

PLOS ONE

The first nation-wide study on facing and solving ethical dilemmas among healthcare professionals in Slovenia

--Manuscript Draft--

**Manuscript Number:**	PONE-D-20-08547
**Article Type:**	Research Article
**Full Title:**	The first nation-wide study on facing and solving ethical dilemmas among healthcare professionals in Slovenia
**Short Title:**	Ethical dilemmas among healthcare professionals in Slovenia
**Corresponding Author:**	Blaž Ivanc, Ph.D.<br>University of Ljubljana - Faculty of Health Sciences / Zdravstvena fakulteta - Univerza v Ljubljani<br>Ljubljana, SLOVENIA
**Keywords:**	health care, ethics, ethics committees, ethical dilemmas, healthcare professionals, health law
**Abstract:**	BACKGROUND. Healthcare professionals (HCPs), patients and families are often faced with ethical dilemmas. The role of healthcare ethics committees (HECs) is to offer support to them. AIM. The primary objective was to study how often HCPs encounter ethical dilemmas. The secondary objective was to identify the main types of ethical dilemmas encountered and how do HCPs solve them. SUBJECTS AND METHODS. We conducted a cross-sectional, survey-based study among HCPs in 14 Slovenian hospitals. A questionnaire was designed and validated for HCPs who were selected by proportional stratified sampling. Data collection took place between April 2015 and April 2016. RESULTS. The final sample size was n = 485 (385 or 79.4% females), response rates for HCPs working in secondary and tertiary level institutions were 45% and 51%, respectively. Three hundred and forty (70.4%) out of 485 HCPs (very) frequently encountered ethical dilemmas. Frequent ethical dilemmas were: waiting times for diagnostics or therapeutic treatment, suboptimal working conditions due to poor interpersonal relations on the ward, preserving patient's dignity and relations between HCPs and patients. Physicians and nurses working in secondary level institutions, compared to their colleagues working in tertiary level institutions, more frequently encountered ethical dilemmas with respect to preserving patient's dignity, patient's information protection, relations between HCPs and patients. In terms of solutions, all HCPs most commonly discuss ethical dilemmas with co-workers (colleagues), and with head of department. According to HCPs, the most important role of HECs is staff education, followed by improving communication, and reviewing difficult ethical cases. CONCLUSIONS. Waiting periods for diagnostics and treatment and suboptimal working conditions due to poor interpersonal relations are considered to be among the most important ethical issues by HCPs in Slovenian hospitals. The most important role of HECs is staff education, improving communication, and reviewing difficult ethical cases.
**Order of Authors:**	Blaž Ivanc, Ph.D.
	Štefan Grosek
	Rok Kučan
	Jon Grošelj
	Ana Borovečki
	Urh Grošelj
	Miha Oražem
	Vanja Erčulj
	Jaro Lajovic
**Additional Information:**	

Powered by Editorial Manager® and ProduXion Manager® from Aries Systems Corporation

Question	Response
**Financial Disclosure**<br><br>Enter a financial disclosure statement that describes the sources of funding for the work included in this submission. Review the submission guidelines for detailed requirements. View published research articles from *PLOS ONE* for specific examples.<br><br>This statement is required for submission and **will appear in the published article** if the submission is accepted. Please make sure it is accurate.<br><br>**Unfunded studies**<br>Enter: *The author(s) received no specific funding for this work.*<br><br>**Funded studies**<br>Enter a statement with the following details:<br>• Initials of the authors who received each award<br>• Grant numbers awarded to each author<br>• The full name of each funder<br>• URL of each funder website<br>• Did the sponsors or funders play any role in the study design, data collection and analysis, decision to publish, or preparation of the manuscript?<br>• **NO** - Include this sentence at the end of your statement: *The funders had no role in study design, data collection and analysis, decision to publish, or preparation of the manuscript.*<br>• **YES** - Specify the role(s) played.<br><br>* typeset	The study was funded as part of the research program Terciar—Project development of the Committee for Medical Ethics University Medical Centre Ljubljana and Clinical Ethics (No: 20140215) of the University Medical Centre Ljubljana in the years 2014–2016.
**Competing Interests**<br><br>Use the instructions below to enter a competing interest statement for this submission. On behalf of all authors, disclose any competing interests that could be perceived to bias this work—acknowledging all financial support and any other relevant financial or non-financial competing interests.<br><br>This statement **will appear in the published article** if the submission is	The authors have declared that no competing interests exist.

accepted. Please make sure it is accurate. View published research articles from *PLOS ONE* for specific examples.<br><br>**NO authors have competing interests**<br><br>Enter: *The authors have declared that no competing interests exist.*<br><br>**Authors with competing interests**<br><br>Enter competing interest details beginning with this statement:<br><br>*I have read the journal's policy and the authors of this manuscript have the following competing interests: [insert competing interests here]*<br><br>* typeset	
**Ethics Statement**<br><br>Enter an ethics statement for this submission. This statement is required if the study involved:<br><br>• Human participants<br>• Human specimens or tissue<br>• Vertebrate animals or cephalopods<br>• Vertebrate embryos or tissues<br>• Field research<br><br>Write "N/A" if the submission does not require an ethics statement.<br><br>General guidance is provided below. Consult the submission guidelines for detailed instructions. **Make sure that all information entered here is included in the Methods section of the manuscript.**	The study was approved by the National Medical Ethics Committee of the Republic of Slovenia on January 1, 2015 (ref. No. of approval 43/01/15 and ref. No. 0120-68/2018/8) The research presented for re-evaluation is observational, all data of included healthcare (HEC) providers were collected in that way that anonymity of included HEC providers was fully obtained in the way that personal information of the HEC providers at the departments cannot be recognized. Therefore the NMEC RS stated that for participation in research and use of necessary data to include them no written informed consent is needed.

**Format for specific study types**<br><br>**Human Subject Research (involving human participants and/or tissue)**<br>• Give the name of the institutional review board or ethics committee that approved the study<br>• Include the approval number and/or a statement indicating approval of this research<br>• Indicate the form of consent obtained (written/oral) or the reason that consent was not obtained (e.g. the data were analyzed anonymously)<br><br>**Animal Research (involving vertebrate animals, embryos or tissues)**<br>• Provide the name of the Institutional Animal Care and Use Committee (IACUC) or other relevant ethics board that reviewed the study protocol, and indicate whether they approved this research or granted a formal waiver of ethical approval<br>• Include an approval number if one was obtained<br>• If the study involved *non-human primates*, add *additional details* about animal welfare and steps taken to ameliorate suffering<br>• If anesthesia, euthanasia, or any kind of animal sacrifice is part of the study, include briefly which substances and/or methods were applied<br><br>**Field Research**<br><br>Include the following details if this study involves the collection of plant, animal, or other materials from a natural setting:<br>• Field permit number<br>• Name of the institution or relevant body that granted permission	
**Data Availability**<br><br>Authors are required to make all data underlying the findings described fully available, without restriction, and from the time of publication. PLOS allows rare exceptions to address legal and ethical concerns. See the PLOS Data Policy and FAQ for detailed information.	Yes - all data are fully available without restriction

A Data Availability Statement describing where the data can be found is required at submission. Your answers to this question constitute the Data Availability Statement and **will be published in the article**, if accepted.<br><br>**Important:** Stating 'data available on request from the author' is not sufficient. If your data are only available upon request, select 'No' for the first question and explain your exceptional situation in the text box.<br><br>Do the authors confirm that all data underlying the findings described in their manuscript are fully available without restriction?	
**Describe where the data may be found in full sentences. If you are copying our sample text, replace any instances of XXX with the appropriate details.**<br><br>• If the data are **held or will be held in a public repository**, include URLs, accession numbers or DOIs. If this information will only be available after acceptance, indicate this by ticking the box below. For example: *All XXX files are available from the XXX database (accession number(s) XXX, XXX.).*<br>• If the data are all contained **within the manuscript and/or Supporting Information files**, enter the following: *All relevant data are within the manuscript and its Supporting Information files.*<br>• If neither of these applies but you are able to provide **details of access elsewhere**, with or without limitations, please do so. For example:<br><br>*Data cannot be shared publicly because of [XXX]. Data are available from the XXX Institutional Data Access / Ethics Committee (contact via XXX) for researchers who meet the criteria for access to confidential data.*<br><br>*The data underlying the results presented in the study are available from (include the name of the third party*	All relevant data are within the manuscript and its Supporting Information files.

and contact information or URL).<br>• This text is appropriate if the data are owned by a third party and authors do not have permission to share the data.<br><br>* typeset	
Additional data availability information:	

Manuscript

Click here to download Manuscript Grosek et al PLOS ONE manuscript_CB-R1-24.03.2020.docx

The first nation-wide study on facing and solving ethical dilemmas among healthcare professionals in Slovenia

Štefan Grosek[1,2,10], Rok Kučan[3], Jon Grošelj[4], Miha Oražem[5], Urh Grošelj[6], Vanja Erčulj[7], Jaro Lajovic[7], Ana Borovečki[8], Blaž Ivanc[9*]

[1]Department of Pediatric Surgery and Intensive Therapy, Division of Surgery, University Medical Centre Ljubljana, Slovenia

[2]Chair of Pediatrics, Faculty of Medicine, University of Ljubljana, Ljubljana, Slovenia

[3]Faculty of Medicine, University of Ljubljana, Ljubljana, Slovenia

[4]Faculty of Theology, University of Ljubljana, Ljubljana, Slovenia

[5]Department of Radiation Oncology, Institute of Oncology Ljubljana, Ljubljana, Slovenia

[6]Department of Pediatric Endocrinology, Diabetes and Metabolic Diseases, University Children's Hospital, UMC Ljubljana, Ljubljana, Slovenia

[7]Rho Sigma Research & Statistics, Ljubljana, Slovenia

[8]Andrija Štampar School of Public Health, School of Medicine, University of Zagreb, Zagreb, Croatia.

[9]Faculty of Health Sciences, University of Ljubljana, Ljubljana, Slovenia

[10]Service of Neonatology, Department of Perinatology, Division of Obstetrics and Gynecology, University Medical Centre Ljubljana

[*]Corresponding author

E-mail: blaz.ivanc@zf.uni-lj.si (BI)

COI: The authors declare that they have no conflict of interest.

Abstract

BACKGROUND. Healthcare professionals (HCPs), patients and families are often faced with ethical dilemmas. The role of healthcare ethics committees (HECs) is to offer support to them. AIM. The primary objective was to study how often HCPs encounter ethical dilemmas. The secondary objective was to identify the main types of ethical dilemmas encountered and how do HCPs solve them. SUBJECTS AND METHODS. We conducted a cross-sectional, survey-based study among HCPs in 14 Slovenian hospitals. A questionnaire was designed and validated for HCPs who were selected by proportional stratified sampling. Data collection took place between April 2015 and April 2016. RESULTS. The final sample size was n = 485 (385 or 79.4% females), response rates for HCPs working in secondary and tertiary level institutions were 45% and 51%, respectively. Three hundred and forty (70.4%) out of 485 HCPs (very) frequently encountered ethical dilemmas. Frequent ethical dilemmas were: waiting times for diagnostics or therapeutic treatment, suboptimal working conditions due to poor interpersonal relations on the ward, preserving patient's dignity and relations between HCPs and patients. Physicians and nurses working in secondary level institutions, compared to their colleagues working in tertiary level institutions, more frequently encountered ethical dilemmas with respect to preserving patient's dignity, patient's information protection, relations between HCPs and patients. In terms of solutions, all HCPs most commonly discuss ethical dilemmas with co-workers (colleagues), and with head of department. According to HCPs, the most important role of HECs is staff education, followed by improving communication, and reviewing difficult ethical cases. CONCLUSIONS. Waiting periods for diagnostics and treatment and suboptimal working conditions due to poor interpersonal relations are considered to be among the most important ethical issues by HCPs in Slovenian hospitals. The most important role of HECs is staff education, improving communication, and reviewing difficult ethical cases.

Introduction

Technological innovations in medicine along with social movements in the United States and later in Europe in 1950’s and 1960’s have brought about new and different ethical challenges in healthcare institutions which invariably involve a tension between HCP[illegible]ersonal values with either institutional, legal, ethical or personal values of others and the society [1-4]. This may finally result, if not properly addressed, in personal moral distress [5, 6] and/or political and legal decision-making conflicts in the most prominent cases [7-10]. In the era of rationalization (rationing), constraints on financial and human resources, and changes in hospital management, these new constraints put even higher pressure on HCP’s values with respect to what is right or wrong [11,12]. Apart from renowned public cases on difficult ethical and mostly legal aspects in the diverse era of medicine [7-10], surveys among HCPs on confrontation with ethical dilemmas and ways of resolving them showed that medical science and clinical medicine professionals have to work together with ethicists, legal institutions, and public domains.

As of December 2019, Medline search query with the keyword “ethical dilemmas” returned around 4,500 hits.

Hospital or institutional ethics committees were first introduced in the United States and later in Europe and other countries. Hospital ethics committees (HECs) have had tremendous impact on recognizing, improving, and resolving diverse ethical dilemmas. In the United States, HECs have existed since the early 1970s [13]. Only later, HECs were introduced in countries all over the Europe as well as globally [14-23].Not only HECs but also other possibilities of resolving ethical dilemmas developed mostly in the Northern parts of Europe where through professional training of HCPs they become facilitators of moral case deliberation among co-workers with whom they work every day (24, 25).

In Slovenia, resolving ethical dilemmas is still in hands of the HECs and no official ethics training is available at the moment for [illegible]Cs. Even in some difficult cases other countries, i.e. with well-established HECs recognized that physicians should be more active when the physicians are faced with situations when people may be affected due to their ignorance (26).

In Slovenia, the first HEC was established at the Faculty of Medicine, University of Ljubljana, in the second half of 1960's after the Helsinki declaration on human research came into effect [27, 29]. It was among the first in Europe and its primary role was to evaluate ethical adequacy of medical research projects by postgraduate students [28]. Also, at the Faculty of Medicine, ethics were being taught since 1948 [29]. However, HECs have only been introduced in the late 1990's when ethics committees were already well established and functioning elsewhere in Europe. Only a small number of committees were actually working on regular basis and not all hospitals had their own ethical committee. Since the very last information, HECs are now established and working regularly in all Slovenian hospitals.

In everyday clinical work, it is of utmost importance that the recommendations of HECs are implemented consistently and in line with other hospital services for maintaining good clinical practice [30-32].

HECs must be evaluated to see, if their work has an impact and which of the envisioned functions (e.g., individual case consultations, education of HCPs, and policy formation) do HECs in fact perform in everyday clinical work [17, 33-36].

A motivation for this study was to evaluate how HCP face ethical dilemmas and how they resolve them. This is the first national survey on HCPs (physicians, nurses, and other profiles of HCPs) working in 14 hospitals (secondary and tertiary level institutions). The primary objective was to study how often HCPs encounter ethical dilemmas. The secondary objective was to identify the main types of ethical dilemmas they are faced with and how do they solve them. We hypothesized that HCPs are frequently faced with ethical dilemmas. Fur-

ther, the type of ethical dilemmas they encounter, the approaches used for ethical education, the awareness of the existence of HECs as well as the extent to which the presence of HECs is considered to really matter for resolving ethical dilemmas are likely to differ among HCPs from different hospitals. A special interest of this study was also to ask whether there are differences between secondary and tertiary health care institutions in terms of what types of ethical dilemmas are most prevalent and how do HCPs solve them.

Methods

Overall Design of the study

We conducted a cross-sectional, survey-based study among HCPs (physicians, nurses and other HCPs') in all ten general hospitals, two special hospitals for treatment of lung diseases, and in both tertiary level university medical centers, that is, altogether in 14 Slovenian hospitals. We prepared a questionnaire form (see Appendix 1) designed for HCPs who were selected by the proportional stratified sampling method.

Study Context and Participants

Based on results from a prior pilot study [37], we expected 60% of HCPs to had faced ethical dilemmas (very) often. To detect the effect with an accuracy of 5% at the significance level α = 0.05 and with 80% power, n = 770 HCPs was required in the study. The expected non-response rate of 30%, increases the sample size by additional n = 230 HCPs.

The inclusion criteria were if the participants were HCPs (physicians, nurses) as well as other HCPs, (laboratory technicians and engineers, radiologic engineers, clinical psychologists, nurse assistants, technicians and engineers of biochemistry, pharmacists, social workers, physiotherapists, respiratory therapists, speech therapists, hygiene technicians, psychologists).

Due to different professional and academic workload and obligations of HCPs and other HCPs between secondary and tertiary level institutions we expect that different ethical dilemmas will be perceived within each groups and subgroups of HCPs and other HCPs.

Proportional stratified sampling was used to select HCPs for the study. The anonymized list of HCPs with their unique IDs was sent from each of the hospitals included in the study. We computed the proportion of HCPs to be included in the sample for each hospital. The employees included in the sample were selected on the basis of simple random sampling (the number of seed selection units in the sample was 02031979). Simple random sampling was performed with the R software package [38] via the call to the function "sample" and with random seed set to the date of the received list of HCPs.

Data collection

Hospitals were included in the study consecutively, one by one. The first hospital included in the study was the UMC Ljubljana. The data collection at the UMC Ljubljana lasted from April to July 2015. The data from the other hospitals were collected after the summer holidays in the autumn and winter of 2015 and the spring of 2016. We chose a simple random sampling method for selecting the employees who meet the criteria for inclusion in the stud[illegible]

The whole list of employees was obtained from the Human Resources Department of each hospital. According to the decision No. 090-59 / 2009 of the Information Commissioner dated July 13, 2009, follows the view that a public employee is not entitled to expect privacy with regard to their names. It follows from the foregoing that the personal information of the employees at the department can be acquired from each employee if they decide to participate in the survey. The list was arranged by employees' surnames in the alphabetical order. We

informed the head and the head nurse of the clinical department of the hospitals by telephone and later by e-mails about the objectives of this research. The questionnaires were delivered to the Secretariat at all clinical departments and services of each hospital personally or by internal mail. Departmental secretaries were asked to distribute the questionnaires to the selected HCPs. In cases of small hospitals, the internal mails were sent directly to HCPs. The responses to the questionnaires were then collected and put in the designated envelopes which were collected after two weeks and moved into a larger box. In this way, we ensured complete anonymity of participants in the survey.

In some hospitals, they disclosed only personal registration numbers and professional profiles of HCPs. Therefore, after we chose eligible HCPs according to their personal registration, the Human Resources Department in each of those hospitals distributed the questionnaire to HCPs by using their personal registration number.

Questionnaire form

The questionnaire was study specific (found in Appendix 1). It consisted of 20 questions, 8 of which were aimed at obtaining demographic information about the respondents.

Questions were divided into three parts. In the first part of the questionnaire, we asked for demographic data (age, gender, information about the profession, workplace, and work experience). In the second part, we aimed at determining how often HCPs are faced with ethical dilemmas and how do they and to estimate how do they encounter ethical dilemmas in the domains listed in the questionnaire in their professional work. We were interested mainly in how they solved the recognized ethical dilemmas and what are the most important area of responsibility of the HECs . The third part consisted of questions about the HEC in their institution. We were interested in finding out the percentage of HCPs that are aware of the existence of the HEC. To four out the 12 questions in the second and third part, the respondents

answered questions by using a five-point Likert scale with frequency labels. In four questions, the respondents were asked to indicate “yes”, “do not know” or “no” as their answer. The remaining 4 were multiple choice questions with an option of an additional written response. The questionnaire was anonymous.

The completion of the questionnaire took about 10 minutes. The questionnaire was accompanied by a text which explained the background and purpose of the study.

Validation and testing the questionnaire

Seven-step approach to questionnaire development as recommended by AMEE guidelines were followed (39):

Step 1: Literature review

Step 2: Interviews / focus groups

Step 3: Synthesize the literature review and interviews

Step 4: Develop items

After first four steps the last three steps were followed with final testing reliability:

5. Establishing **face validity**: different experts (physicians, members of CMEs, lawyers and lay persons) evaluated the content of the questionnaire. Questionnaire was changed accordingly. It was checked further for wording, possible double-barrelled, confusing and misleading questions by two psychometricians.

6. **Pilot test**: the pilot questionnaire was given and answered by 35 health-care professionals of different occupational profiles. Participants commente[illegible]e questionnaire, pointed possible difficulties they had with understanding or answering certain questions. On the basis of comments and results of the statistical analysis, some of the items were dropped from the final questionnaire.

7. **Validity:** Exploratory factor analysis on the whole Slovene sample showed all the items measuring frequency of ethical dilemmas loaded substantially (loadings > 0.50) on the same factor. The validity of the questionnaire was assessed further by investigating correlations between general question on ethical dilemma frequency and questions on each specific dilemma frequency. Correlation coefficients varied between 0.27 and 0.52 and were all statistically significant ($p < 0.001$).

Reliability: the questionnaire exhibits high internal consistency (Cronbach α = 0.93). Test-retest reliability was not assessed to ensure/guarantee complete respondent anonymity. The later was crucial for the validity of the obtained results.

Statistics

Mean and standard deviations were calculated for continuous and frequencies and percentages for categorical variables. Answers on Likert-type 5-point scale items dichotomized in two categories. Answers “frequently” and “very frequently” formed the first and all the other the second answer category. The share of HCPs (very) frequently encountering an ethical dilemma was calculated and the 95% CI was obtained using the bootstrap method. The relationship between a HCP’s profile and the binary outcome variables were analyzed using mixed-effect logistic regression model to account for clustering effect of individuals within hospitals. A mixed-effect linear regression model was used to analyze the relationship between a worker’s profile and numerical outcome variables. The significance level threshold was set to $\alpha = 0.05$. The analysis was performed using SPSS v. 23.

Ethical Considerations

The study was approved by the National Medical Ethics Committee of the Republic of Slovenia on January 1, 2015 (No. of approval 43/01/15 and ref. No 0120-68/2018/8). The research presented for re-evaluation is observational; all data of included healthcare (HEC) providers were collected in that way that anonymity of included HEC providers was fully obtained in the way that personal information of the HEC providers at the departments cannot be recognized. Therefore the NMEC RS stated that for participation in research and use of necessary data to include them no written informed consent [illegible]s needed.

Results

The Questionnaires were sent to the following 14 hospitals in Slovenia: Maribor (141), Topolščica (10), Golnik (21), Jesenice (34), Izola (40), Nova Gorica (39), Novo Mesto (48), Brežice (19), Ptuj (25), Murska Sobota (40), Slovenj Gradec (38), Celje (86), Trbovlje (14), and Ljubljana (44[illegible] The response rates by hospital were 44%, 80%, 62%, 26%, 28%, 41%, 42%, 68%, 80%, 85%, 37%, 28%, 57% and 52%, respectively. The response rates in the secondary and tertiary level institutions were 45% and 51[illegible] respectively. The final sample size was n = 485.

Characteristics of participating HCPs

The demographic characteristics of HCPs' participating in the study are given in Table 1. The sample consisted of 385 (79.4%) females. The mean (standard deviation) age, employment period and years of employment period in the current hospital were 40.9 (10.6), 18.8 (11.4) and 16.6 (11.1), respectively. The sample consisted of 76 (15.7%) physicians, 320 (66%) nurses, and 89 (18.4%) other HCP[illegible]

Table 1. Sample characteristics of healthcare professionals (HCPs) (n = 485).

	**n (%)**
**GENDER**	
Male	100 (20.6)
Female	385 (79.4)
**Mean age (SD; n = 481)**	40.9 (10.6)
**Mean years of employment period (SD; n = 484)**	18.8 (11.4)
**Mean years of employment period in current hospital (SD; n = 484)**	16.6 (11.1)
**Religion (n = 480)**	
Religious	364 (75.8)
Not religious	56 (11.7)
**TYPE OF INSTITUTION (n = 471)**	
Secondary level	211 (44.8)
Tertiary level	260 (55.2)

**PROFILES OF HCPs**	
Physicians	76 (15.7)
Nurses	320 (66)
Other HCPs	89 (18.4)
**HOSPITAL UNIT (n = 484)**	
Reception clinic	21 (4.3)
Clinic	75 (15.5)
Emergency wards	24 (5)
Hospital wards	184 (38)
Intensive care units	50 (10.3)
Diagnostic wards	36 (7.4)
Surgical wards	53 (11)
Elsewhere	41 (8.5)

HCPs – health care professionals. Other HCPs - laboratory technicians and enginers, radiologic enginers, clinical psychologists, nurse assistants, technicians and enginers of biochemistry, pharmacists, social workers, physiotherapists, respiratory therapists, speech therapists, hygiene technicians, psychologists.

The share of the profiles of HCPs included in the sample statistically significantly differs from the share in the population (p = 0.036). In our sample, physicians are underrepresented (23 more physicians were expected in the sample of the given size) whereas nurses are overrepresented (26 less nurses was expected in the sample of the given size). There were 211 (44.8%) HCPs from the secondary level institution and 260 (55.2%) HCPs from the tertiary level institution included in the sample. The distribution of HCPs by the institution type does not differ statistically significantly from the one in the population (p = 0.183).

Out of 483 HCPs that answered the questions on ethical dilemmas, 340 (70.4%; 95% CI: 66.7 – 74.5%) (very) frequently encountered an ethical dilemma during work (Table 2). The percentages of physicians, nurses, and other HCPs (very) often encountering an ethical dilemma are 90.8%, 67.7%, and 62.5%, respectively. Logistic regression showed that physicians have ~6-times higher odds for facing an ethical dilemma compared with other HCPs (OR = 5.8; 95% CI: 2.3-14.5; Table 2). The odds for frequent encounter with an ethical dilemma for nurses are comparable to those of other HCPs (p = 0.601). From all ethical dilem-

mas, most frequently encountered ethical dilemmas by the largest share of HCPs are: waiting periods for diagnostics or therapeutic treatment (40%), followed by suboptimal working conditions due to poor interpersonal relations on the ward (32.1%), preserving patient's dignity (30.1%), and relations between health care professionals and patients (or their legal guardians) (29.1%). Frequent ethical dilemmas recognized by the largest share of physicians, nurses, and other HCPs are presented in Table 2.

Table 2. The percentages of (very) frequently encountered ethical dilemmas and association between type of HCPs and ethical dilemmas.

	Physicians (n = 76)		Nurses (n = 320)		Other HCPs (n = 89)		All HCPs (n = 485)		Physicians		Nurses	
	n (%)	R	n (%)	R	n (%)	R	n (%)	R	OR (95 CI)	p-value	OR (95 CI)	p-value
**Ethical dilemma**	**69 (90.8)**	**76**	**216 (67.7)**	**319**	**55 (62.5)**	**88**	**340 (70.4)**	**483**	**5.8 (2.3; 14.5)**	**< 0.001**	**1.1 (0.7; 1.9)**	**0.601**
**Waiting periods for diagnostics or therapeutic treatment**	53 (69.7)	76	113 (36.2)	312	21 (26.6)	79	187 (40)	467	6.8 (3.3; 14.1)	**< 0.001**	1.7 (1; 3)	0.073
**Suboptimal working conditions due to poor interpersonal relations on the ward**	40 (52.6)	76	94 (29.9)	314	18 (21.7)	83	152 (32.1)	473	4.2 (2; 8.6)	**< 0.001**	1.6 (0.9; 2.9)	0.12
**Preserving patient's dignity**	23 (30.7)	75	95 (30.4)	313	23 (28.8)	80	141 (30.1)	468	1.2 (0.6; 2.6)	0.614	1.1 (0.6; 2)	0.782
**Relations between HCPs and the patients (or his/her relatives)**	25 (32.9)	76	91 (29.2)	312	20 (25.3)	79	136 (29.1)	467	1.5 (0.7; 3.1)	0.272	1.2 (0.7; 2.1)	0.594
**Recognizing patient's best interests**	29 (38.7)	75	73 (23.5)	310	25 (31.3)	80	127 (27.3)	465	1.5 (0.8; 3.1)	0.242	0.7 (0.4; 1.2)	0.189
**Patient information protection**	15 (19.7)	76	85 (27.1)	314	25 (30.9)	81	125 (26.5)	471	0.5 (0.2; 1.1)	0.081	0.8 (0.5; 1.5)	0.529
**End-of-life treatment withdrawal**	36 (49.3)	73	78 (25.2)	309	5 (6.3)	79	119 (25.8)	461	15.8 (5.6; 44.6)	**< 0.001**	5.3 (2; 13.7)	**< 0.001**
**New modes of treatment and diagnostic procedures**	20 (26.3)	76	67 (21.5)	311	10 (12.7)	79	97 (20.8)	466	2.5 (1.04; 5.8)	**0.039**	2 (1; 4.2)	0.064
**Allocation of limited resources**	28 (37.3)	75	53 (17.2)	309	14 (17.3)	81	95 (20.4)	465	2.9 (1.4; 6.2)	**0.006**	1 (0.5; 1.9)	0.917

**Lack of response to adverse events in the patient management**	25 (32.9)	76	50 (16.2)	309	8 (9.9)	81	83 (17.8)	466	4.6 (1.9; 11.4)	< **0.001**	1.7 (0.8; 3.9)	0.18
**Insufficient availability of palliative care**	20 (27)	74	59 (19)	311	2 (2.6)	78	81 (17.5)	463	18.1 (3.9; 83.7)	< **0.001**	10.4 (2.4; 45)	**0.002**
**Patient's consent to participate in a diagnostic or therapeutic treatment**	14 (18.7)	75	64 (20.4)	313	8 (10.1)	79	86 (18.4)	467	2.2 (0.8; 5.6)	0.113	2.3 (1.1; 5.2)	**0.037**
**Disagreement with individual's profes-sional work**	18 (23.7)	76	55 (17.7)	310	7 (8.6)	81	80 (17.1)	467	3.5 (1.3; 9.3)	**0.011**	2.5 (1.1; 6)	**0.034**
**Learning on patients**	10 (13.2)	76	51 (16.3)	312	14 (17.7)	79	75 (16.1)	467	0.7 (0.3; 1.8)	0.503	0.9 (0.4; 1.8)	0.728
**Patient's right to refuse treatment**	8 (10.5)	76	48 (15.4)	311	2 (2.6)	77	58 (12.5)	464	4.4 (0.9; 21.7)	0.07	7.2 (1.7; 30.7)	**0.008**
**Social inequality or withdrawal basic healthcare insurance**	12 (15.8)	76	34 (10.9)	311	2 (2.5)	79	48 (10.3)	466	7 (1.5; 32.9)	**0.013**	4.5 (1.1; 19.5)	**0.042**
**Involuntary hospitalization**	8 (10.7)	75	33 (10.6)	312	2 (2.6)	77	43 (9.3)	464	4.4 (0.9; 21.6)	0.07	4.5 (1; 19.4)	**0.043**
**Biomedical research**	4 (5.3)	75	21 (6.8)	308	5 (6.5)	77	30 (6.5)	460	0.8 (0.2; 3)	0.687	1.1 (0.4; 3)	0.919
**Organ transplantation**	4 (5.5)	73	20 (6.5)	306	2 (2.5)	79	26 (5.7)	458	2.2 (0.4; 12.6)	0.374	3 (0.7; 13.4)	0.145
**Refusal of vaccines**	4 (5.4)	74	19 (6.1)	312	3 (3.8)	78	26 (5.6)	464	1.3 (0.3; 6.1)	0.753	1.6 (0.4; 5.6)	0.482

Results of univariate mixed-effect logistic regression with other HCPs as reference category. HCPs – healthcare professionals, R – number of respondents, OR – odds ratio adjusted for hospital, CI – confidence interval.

Compared to other HCPs, physicians have higher odds of encountering ethical dilemmas regarding new modes of treatment and diagnostic procedures, end-of-life treatment withdrawal, suboptimal working conditions due to poor interpersonal relations on the ward, disagreement with an individual's professional work, lack of response to adverse events in patient management, social inequality or withdrawal of patient rights from base healthcare insurance, allocation of limited resources, insufficient availability of palliative care, and waiting times for diagnostics or therapeutic treatment (Table 2).

Compared to other HCPs, nurses have statistically significantly higher odds of encountering ethical dilemmas regarding end-of-life treatment withdrawal, disagreement with an individual's professional work, social inequality or withdrawal of patient rights from base healthcare insurance, patient's consent to participate in a diagnostic or therapeutic treatment (Table 2).

Ethical dilemmas among HCPs working in secondary and tertiary level institutions

Physicians and nurses working in secondary level institutions, compared to their colleagues working in tertiary level institutions, more frequently encounter ethical dilemmas regarding preserving patient's dignity, patient's information protection, and relations between HCPs and patients (Fig 1). In addition, physicians working in secondary level, compared to those working in tertiary level institutions, more often encountered ethical dilemmas pertaining to patient's consent to participate in a diagnostics and therapeutic treatment. Nurses working at the secondary level institution were more often faced with an ethical dilemma of recognizing the patient's best interest compared to nurses working at the tertiary level institutions. Physicians and nurses working in secondary level institutions have higher odds for more fre-

quent encounter with abovementioned ethical dilemmas in comparison to physicians and nurses working in tertiary level institution (data not shown).

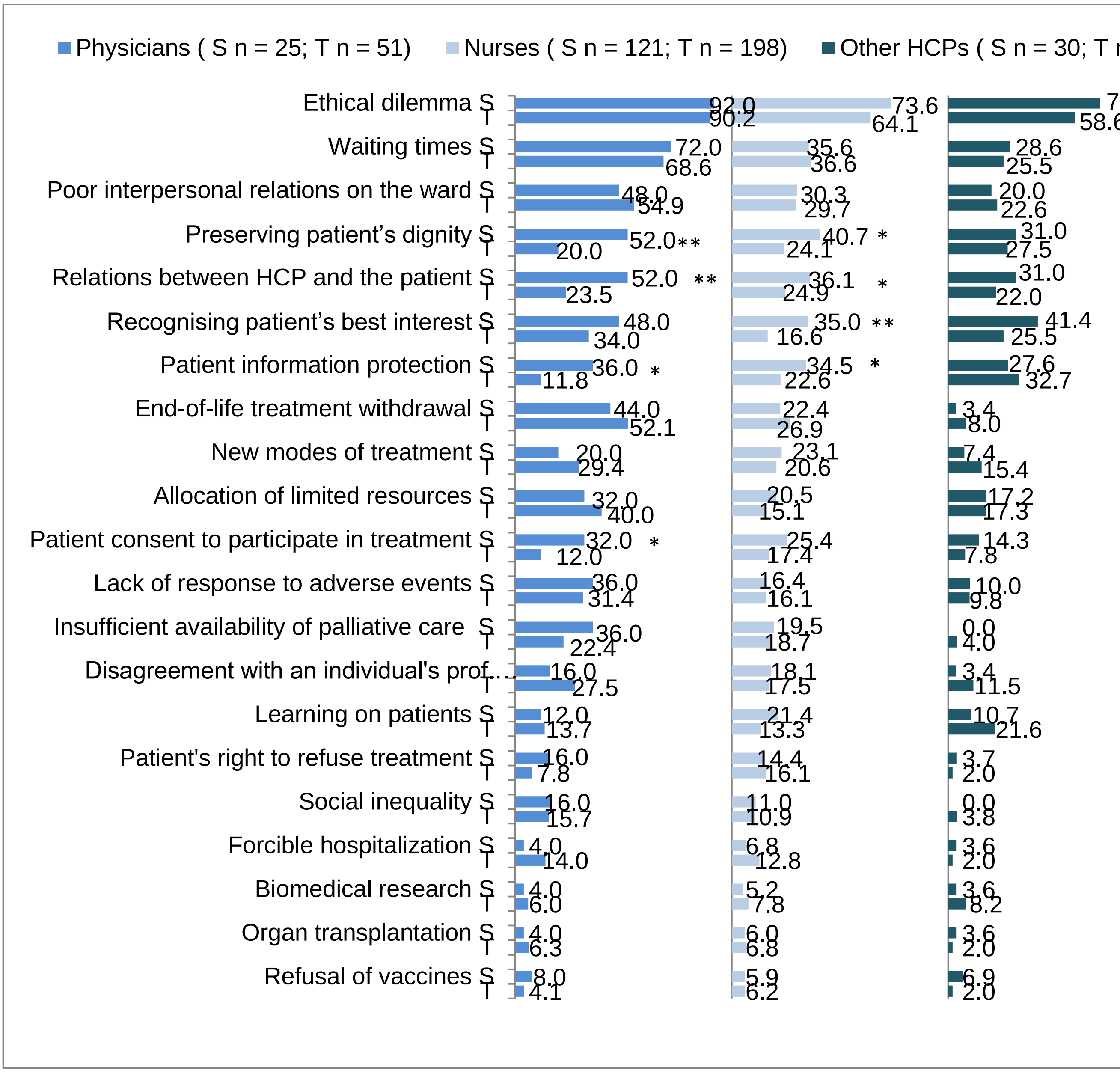

Fig 1. Frequent ethical dilemmas by the type of institution. S – secondary level institution, T – Tertiary Level Institution, HCPs – Healthcare Professionals, MCS – Medical Chamber of Slovenia.

Who do HCPs consult with when confronted with an ethical dilemma

Table 3 shows the results on who do HCPs consult with when faced with an ethical dilemma in their work. Most frequently they discuss it with colleagues (94.2%) and/or with the head of the department (55.2%). Physicians most commonly discuss ethical dilemmas with colleagues (90.8 %), with the head of the department (75%), or they call a medical council meeting (47.4%). Nurses and other HCPs most commonly discuss ethical dilemmas with colleagues (96.3%, 89.5%, respectively) and with the head of the department (48.8%, 61.6%, respectively). Physicians have higher odds (OR = 96.9; 95% CI: 12.4–757.2) of calling a medical council meeting or to consult the issue with an HEC (OR = 17.8; 95% CI: 3.9–82.3) in comparison to other HCPs.

Compared to other HCPs, nurses have lower odds (OR = 0.6; 95% CI: 0.3–0.9) of discussing ethical dilemmas with the head of the department or of deciding alone (OR = 0.3; 95% CI: 0.2–0.7), but higher odds of discussing ethical dilemmas with colleagues (OR = 3.2; 95% CI: 1.2–8.1).

Table 3. Association between types of HCPs and ways of dealing when faced with ethical dilemmas.

	Physicians (n = 76)	Nurses (n = 320)	Other HCPs (n = 86)	All HCPs (n = 482)	Physicians		Nurses	
	n (%)	n (%)	n (%)	n (%)	OR (95 CI)	p-value	OR (95 CI)	p-value
**Discuss with co-workers**	69 (90.8)	308 (96.3)	77 (89.5)	454 (94.2)	1.4 (0.5; 4.2)	0.556	3.2 (1.2; 8.1)	**0.017**
**Discuss with head of the department**	57 (75)	156 (48.8)	53 (61.6)	266 (55.2)	2 (1; 4)	0.057	0.6 (0.3; 0.9)	**0.029**
**Decide on my own**	13 (17.1)	24 (7.5)	14 (16.3)	51 (10.6)	1.1 (0.4; 2.5)	0.902	0.3 (0.2; 0.7)	**0.006**
**Call a medical council meet-ing**	36 (47.4)	7 (2.2)	1 (1.2)	44 (9.1)	96.9 (12.4; 757.2)	**< 0.001**	2 (0.2; 16.6)	0.534
**Consult the hospital ethics committee**	19 (25)	10 (3.1)	2 (2.3)	31 (6.4)	17.8 (3.9; 82.3)	**< 0.001**	1.6 (0.3; 7.5)	0.561
**Resolve the issue in family circles**	9 (11.8)	11 (3.4)	4 (4.7)	24 (5)	2.6 (0.7; 9.5)	0.146	0.5 (0.2; 1.8)	0.313
**Consult with patient's legal representative**	3 (3.9)	9 (2.8)	1 (1.2)	13 (2.7)	3.3 (0.3; 33.2)	0.32	2 (0.2; 16.7)	0.532
**Resolve the issue with medi-ation**	3 (3.9)	10 (3.1)	0 (0)	13 (2.7)				
**Consult the hospital chap-**	1 (1.3)	5 (1.6)	0 (0)	6 (1.2)				

**lain**								
**Consult the national ethics committee**	4 (5.3)	0 (0)	0 (0)	4 (0.8)				
**Consult the Human Rights Ombudsman**	2 (2.6)	1 (0.3)	1 (1.2)	4 (0.8)	2 (0.2; 24.5)	0.577	0.2 (0.01; 4.2)	0.333
**Consult the Committee for Legal and Ethical Issues of the Medical Chamber of Slovenia**	0 (0)	1 (0.3)	1 (1.2)	2 (0.4)			0.3 (0; 4.4)	0.358
**Contact the media**	0 (0)	0 (0)	1 (1.2)	1 (0.2)				

Results of univariate mixed-effect logistic regression with other HCPs as reference category. HCPs – healthcare professionals, OR – odds ratio adjusted for hospital, CI – confidence interval.

Fig 2 shows the results on reactions to situations involving ethical dilemmas per each group of HCPs working in secondary and tertiary level institutions.

Physicians in secondary level institutions have higher odds (OR = 5.1; 95% CI: 1.1–22.3) of resolving ethical dilemmas in HCPs' family circle and lower odds (OR = 0.1; 95% CI: 0.01–0.6) of discussing with an HEC.

Nurses in secondary level institutions have higher odds (OR = 2.2; 95% CI: 1.4–3.5) of discussing ethical dilemmas with the head of the department and for resolving dilemma through mediation (OR = 4; 95% CI: 1–15.6) compared to nurses working in tertiary level institutions.

Other HCPs working in secondary level institutions have higher odds (OR = 3.4; 95% CI: 1.2–9.7) of discussing ethical dilemma with the head of the department and for deciding alone (OR = 11; 95% CI: 2.8–43.9) compared to those working in tertiary level institutions.

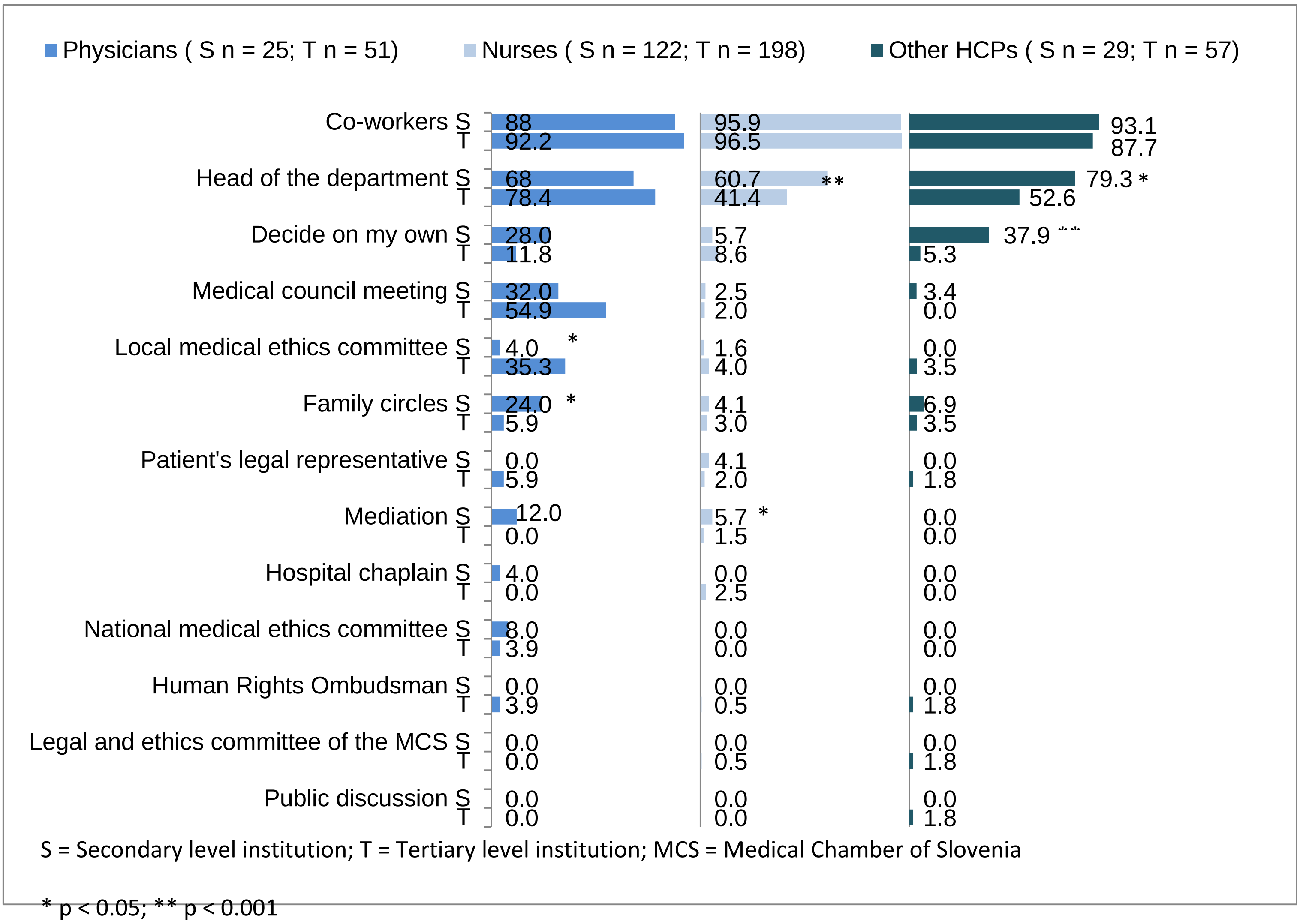

Fig 2. Ways of dealing to ethical dilemmas by type of institution. S – secondary level institution, T – tertiary level institution, HCPs – healthcare professionals, MCS – Medical chamber of Slovenia.

In the investigated groups of HCPs, the highest share of HCPs who are unaware of standard procedures for solving ethical dilemmas was found among physicians (30.7%) compared to nurses (13.2%) and other HCPs (6.7%). From those who answered with “yes of being aware” or “no” for being unaware, the odds of answering “yes” are lower for physicians (OR = 0.2; 95% CI: 0.1–0.6) compared to other HCPs (Table 4).

The odds of not discussing ethical dilemmas are higher for physicians than other HCPs (OR = 2.9; 95% CI: 1.2 – 7.1), but not for nurses compared to other HCPs (p = 0.453).

Table 4. Association between types of HCPs and standard procedures for solving ethical dilemmas or presence of an undiscussed ethical dilemma.

	Physicians	Nurses	Other HCPs	All HCPs	Physicians		Nurses	
					OR (95 CI)	p-value	OR (95 CI)	p-value
**Standard procedures for resolving ethical dilemmas**								
Yes of being aware of	21 (28)	106 (33.3)	26 (29.2)	153 (31.7)	0.2 (0.1; 0.6)	**0.004**	0.6 (0.2; 1.6)	0.288
No for being una-ware of	23 (30.7)	42 (13.2)	6 (6.7)	71 (14.7)	1		1	
Don't know	31 (41.3)	170 (53.5)	57 (64)	258 (53.5)	-	-	-	-
**Undiscussed ethical dilemma**								
Yes	25 (32.9)	63 (19.7)	12 (13.6)	100 (20.7)	2.9 (1.2; 7.1)	**0.019**	1.3 (0.6; 2.9)	0.453
No	36 (47.4)	172 (53.9)	45 (51.1)	253 (52.4)	1		1	
Don't know	15 (19.7)	84 (26.3)	31 (35.2)	130 (26.9)	-	-	-	-

Results of univariate logistic regression with other HCPs as reference category. HCPs – healthcare professionals, OR – odds ratio adjusted for hospital, CI – confidence interval.

Considering all HCPs (n = 483), most common ways of learning about medical ethics are: during educational system or university (24%), learning from more experienced peers

(21.3%), and learning at workshops (20.3%). No differences were found between HCPs. The majority (88.7%) of HCPs believe that regular formal education about ethics in medicine is required for hospital staff. We found no differences between working profiles.

That the role of HECs is important believes 84.6% of HCPs, namely, 79.7% of physicians, 84.9% of nurses, and 87.6% of other HCPs. No statistically significant differences in opinion on the importance of HECs between groups of HCPs could be found (Table 5). About half (51.4%) of HCPs is familiar with existence of an ethics committee in their hospital. There were no statistically significant differences in the shares of physicians, nurses, and other HCPs that are aware of ethics committees in their hospital (Table 5). More than one third (36.5%)of all HCPs know they can consult the HEC. There were no statistically significant differences in the shares of physicians, nurses, and other HCPs in terms of their familiarity with the option to consult HECs (Table 5).

Table 5. Relationship between types of HCPs and the perceived importance of HEC, awareness of its existence, or awareness of the option to consult the HEC.

	Physicians	Nurses	Other HCPs	All HCPs	Physicians		Nurses	
					OR (95% CI)	p-value	OR (95% CI)	p-value
**Importance of the role of medical ethic committee**								
Weaker agreement	15 (20.3)	48 (15.1)	11 (12.4)	74 (15.4)				
Stronger agreement	59 (79.7)	270 (84.9)	78 (87.6)	407 (84.6)	0.6 (0.3; 1.5)	0.28	0.8 (0.4; 1.7)	0.606
**Existence of ethics committee**								
Yes	42 (56)	165 (51.7)	40 (46)	247 (51.4)	0.4 (0.1; 1.9)	0.23	1.1 (0.3; 3.4)	0.908
No	9 (12)	19 (6)	4 (4.6)	202				

				(42)				
Don't know	24 (32)	135 (42.3)	43 (49.4)	32 (6.7)	-	-	-	-
**Option of consultations for staff***								
Yes	27 (52.9)	84 (36.2)	14 (23.7)	125 (36.5)	2.1 (0.5; 8.9)	0.313	1.7 (0.5; 5.7)	0.357
No	5 (9.8)	25 (10.8)	5 (8.5)	182 (53.2)				
Don't know	19 (37.3)	123 (53)	40 (67.8)	35 (10.2)	-	-	-	-

Results of univariate logistic regression with other HCPs as reference category. HCPs – healthcare professionals, OR – odds ratio adjusted for hospital, CI – confidence interval, * – number of respondents = 342; physicians n = 51, nurses n = 232 and other HCPs n = 5.

Number of consultations with HECs in 2014 was 0–2 for physicians, 0–4 for nurses, and 0–1 for other HCPs. Physicians have higher odds (OR = 5.3; 95% CI: 1.6–17.6) for having consulted with an HEC in 2014 compared to other HCPs (p = 0.007).

None of the physicians or nurses and one of the other HCPs working in secondary level hospital consulted with an HEC in 2014. Among the physicians working in a tertiary level institution, 15 (33.3%) consulted with HECs in 2014. The corresponding number for nurses is 12 (6.9%) and 3 (6.4%) for other HCPs.

According to all HCPs, the most important role of HECs is healthcare staff education (47.1%), followed by improving communication (41.1%), and review of difficult cases (35.1%). Physicians, compared to other HCPs, gave higher importance to reviewing of difficult cases (OR = 2; 95% CI: 1.04–3.8), preparation of guidelines or protocols (OR = 2.8; 95% CI: 1.3–5.8), legal protection of physicians in decision-making process (OR = 18.4; 95% CI: 4.1–82.8), and allocation of limited resources (OR = 13.5; 95% CI: 1.6–111.6). On the other

hand, they attribute lower importance to improving communication (OR = 0.3; 95% CI: 0.2–0.6) or conflict resolution (OR = 0.2; 95% CI: 0.1–0.6). Compared to other HCPs, nurses attribute lower importance to improvement of quality of healthcare (OR = 0.5; 95% CI: 0.3–0.9) (Table 6).

Table 6. Association between types of HCPs and the role of HECs.

	Physicians (n = 76)	Nurses (n = 320)	Other HCPs (n = 89)	All HCPs (n = 485)	Physicians		Nurses	
					OR (95% CI)	p-value	OR (95% CI)	p-value
**Education of HCPs**	31 (40.8)	151 (47.5)	45 (51.1)	227 (47.1)	0.6 (0.3; 1.2)	0.148	0.8 (0.5; 1.4)	0.493
**Improving communication**	16 (21.1)	139 (43.7)	43 (48.9)	198 (41.1)	0.3 (0.2; 0.6)	**0.001**	0.9 (0.5; 1.4)	0.586
**Review of difficult cases**	37 (48.7)	103 (32.4)	29 (33)	169 (35.1)	2 (1.04; 3.8)	**0.037**	1 (0.6; 1.6)	0.932
**Conflict resolution**	7 (9.2)	103 (32.4)	27 (30.7)	137 (28.4)	0.2 (0.1; 0.6)	**0.003**	1.1 (0.7; 1.9)	0.669
**Moral support for HCPs**	14 (18.4)	96 (30.2)	18 (20.5)	128 (26.6)	0.9 (0.4; 2.1)	0.86	1.6 (0.9; 3)	0.109
**Ethical consultations for employees (in session form)**	20 (26.3)	69 (21.7)	22 (25)	111 (23)	1 (0.5; 2.1)	0.961	0.9 (0.5; 1.5)	0.621
**Improving the quality of healthcare**	13 (17.1)	55 (17.3)	23 (26.1)	91 (18.9)	0.6 (0.3; 1.3)	0.17	0.5 (0.3; 0.9)	**0.023**
**Developing guidelines or protocols**	27 (35.5)	48 (15.1)	15 (17)	90 (18.7)	2.8 (1.3; 5.8)	**0.008**	0.9 (0.5; 1.8)	0.788
**Ethical consultations in the wards (at the**	10 (13.2)	40 (12.6)	9 (10.2)	59 (12.2)	1.2 (0.4; 3.1)	0.764	1.2 (0.5; 2.6)	0.67

**bedside)**								
**Support of patients in giving them a stronger voice in decision making**	5 (6.6)	45 (14.2)	12 (13.6)	62 (12.9)	0.5 (0.2; 1.5)	0.206	1.1 (0.6; 2.3)	0.746
**Legal protection of physicians in decision making**	23 (30.3)	26 (8.2)	2 (2.3)	51 (10.6)	18.4 (4.1; 82.8)	**< 0.001**	3.7 (0.9; 16.3)	0.079
**Assessment upon introduction of novel treatment modes**	9 (11.8)	23 (7.2)	12 (13.6)	44 (9.1)	0.8 (0.3; 2.1)	0.639	0.5 (0.2; 1.1)	0.088
**Counselling hospital management staff**	3 (3.9)	23 (7.2)	2 (2.3)	28 (5.8)	1.7 (0.3; 10.7)	0.567	3.7 (0.8; 16.3)	0.082
**Allocation of limited resources**	9 (11.8)	12 (3.8)	1 (1.1)	22 (4.6)	13.5 (1.6; 111.6)	**0.016**	3.7 (0.5; 29.5)	0.212
**Other**	0 (0)	2 (0.6)	0 (0)	2 (0.4)	-	-	-	-

Results of univariate logistic regression with other HCPs as reference category. HCPs – healthcare professionals, OR – odds ratio adjusted for hospital, CI – confidence interval.

Discussion

This is the first national survey among healthcare professionals (HCPs; physicians, nurses, and other HCP profiles) from 14 hospitals (secondary and tertiary level institutions) examining ethical dilemmas HCPs are confronted with in every day professional practice, how they solve them, how they use hospital ethics committees (HECs), and what is their opinion in resolving their ethical dilemmas. This study reveals several important findings.

First, all HCPs that participated in the study encountered ethical dilemmas (very) often (70.4%). However, among physicians the share rose up to 90.8% whereas the shares of nurses and other HCPs who encountered ethical dilemmas were 67.7% and 62.5%, respectively. This resulted in physicians having 6 times higher odds for facing ethical dilemmas compared to other HCPs. The cause for this result might be the fact that Physicians Practitioners Act endorse physicians as the solely responsible person for medical activities whether the legislature failed to regulate the professional activity of the nurses and other HCPs (40). No statistically significant differences were found between nurses and other HCPs, as well as between physicians working either in secondary and tertiary level institutions. The share of ethical dilemmas among Slovenian HCPs is comparable with other studies which showed that between 60% and 90% of HCPs encountered various ethical dilemmas in their work [13, 32, 41-43]. Similarly to present results, several international studies also reported that apart from physicians, nurses and other HCPs may also encounter different ethical dilemmas [42, 44-4247]. The demographic characteristics of our HCPs showed that the majority of our participants are females (79,5%) because we included all HCPs in the study where the majority among nurses and physicians are females. In our study sample, physicians are underrepresented and nurses are overrepresented whereas the distribution among secondary and tertiary level institutions did not statistically significant differ from the one in the population. In a previous study performed on a sample from a tertiary level hospital [37], we observed that 60% of HCPs encountered an ethical dilemma while in our study a higher percentage of respondents (70.4%) encountered ethical dilemmas frequently. We collected data from all types of hospital workplaces, that is, from reception clinic, clinic, emergency wards, hospital wards, intensive care units, diagnostic wards, and surgical wards as well as in other workplaces not mentioned previously. This gave us a good insight into a whole array of ethical dilemmas which may confront different profiles of HCPs. Other studies mostly targeted only one or two different pro-

files of HCP[illegible]uVal et al. developed a questionnaire based on a review of the ethics consultation and later included a cognitive method to prepare the questionnaire. The same questionnaire was then used also by Hurst and Sorta-Bilajac to study ethical issues in clinical practice [13,-38-43]. DuVal et al. found that general internists among surveyed American physicians (general internists, oncologists, and critical care specialists) most commonly reported dilemmas regarding end-of-life decision-making, patient autonomy, justice, and conflict resolution [13]. Among different ethical dilemmas, end-of-life decisions and patient autonomy were often referred for consultation, while dilemmas about justice, such as lack of insurance or limited resources, were rarely referred. More knowledgeable and experienced physicians in ethics are significantly more likely to request an ethics consultatio[illegible]n addition, studyof duVal et al also revealed that 41% of physicians express some hesitation about using ethics consultation. This results concur with ours where we found that 35.3% of physicians working in the tertiary level hospitals would discuss with hospital ethical committee compared to only 4% of them in secondary level hospitals when facing with ethical dilemma (p=0.01[illegible]No differences were found for nurses and other HCPs. Therefore we can draw a conclusion that not only physicians in the secondary level hospitals but also nurses and other HCPs in secondary and tertiary level hospitals are probably not enough aware of presence and therefore using hospital ethics committees when facing with ethical dilemmas. More education is probably needed. Hurst et al. performed a study among general internists in Norway, Switzerland, Italy, and the UK and found that uncertain or impaired decision-making capacity, disagreement among caregivers and limitation of treatment at the end-of-life were their the most frequent ethical dilemmas encountered [43]. The third study employing the same questionnaire was performed among Croatian physicians and associated nurses at the University Hospital Rijeka and yielded similar results (41-43). The results from those three studies show that some of the raised questions are very similar or identical to ours, however, with some important differences. Among the most important ethical dilemmas in their studies, and ours as well, were ethical dilemmas regarding the end-of-life decision and relationships between HCPs, but the two most important dilemmas in our study which were not included in others studies were “waiting periods for diagnostics and care of the patients” and “interpersonal relationships on the ward” which were reported by 69.7% and 52.6% of physicians and much less likely in nurses and other HCPs. Because euthanasia and assisted suicide are legally not allowed in Slovenia, we did not pose such questions in our questionnaire. If we suppose that waiting periods in Slovenia are probably not only due to shortage of healthcare personnel but also due to a centrally planned model of healthcare still governed by a single national medical insurance company, it becomes clear

that lack of financial support for all diagnostics, operations, and therapeutic treatments plays a major role as well. Hospitals are therefore allowed to provide only planned and agreed healthcare diagnostics and procedures and nothing more, which is the source of great ethical dilemmas for Slovenian HCPs.

HCPs encounter different types of ethical dilemmas to varying extents depending on the group of HCPs they belong to. While waiting periods, interpersonal relationships on the ward, and end-of-life treatment are most common among physicians, care for patient’s dignity, besides the first two listed among physicians, are encountered by nurses the most. Among other HCPs, recognizing patient’s best interests, patient information protection, and care for patient’s dignity are the three most important ethical dilemmas. What we can derive from this answer is that we should have conducted a new study in which we would take questions on this issue found. One of the possible answers would be: a.) that the other HCPs have access to large amount of data, information when they take care of the patients specifically to their profession and that seems to them the most important, b.) that nobody teach them how to manage and interpret many personal information they came to their ears.

However, no differences were found for any of the ethical dilemmas between secondary and tertiary level institutions for other HCPs. Waiting periods and interpersonal relationships on the ward between secondary and tertiary levels of institutions for all HCPs did not differ significantly among physicians and nurses, which clearly shows that this is a major problem in Slovenian hospitals and that lack of professionalism among physicia[illegible]s one of great problems which leads to poor interpersonal relationships on the ward [48]. Among Bulgarian physicians, predominant dilemmas included relationships with patients and relatives (76.8%) and team work (67.6%) followed by end-of-life issues (31.5%) [49]. Nurses and other HCPs, with the primary responsibility of caring for the patient, are somewhat more receptive to the dilemmas associated with the patient's well-being and dignity. Similarly, Norberg et al. observed that nurses are more emotionally involved in ethical dilemmas concerning patient care compared to physicians as was observed by Norberg et al, too [45-50].

Higher shares of physicians and nurses in the secondary level institutions experienced ethical dilemmas regarding care for patient’s dignity, relationship between HCPs and patients, and patient information protection. One of the reasons for higher shares among HCPs working in secondary levels institutions is due to the fact that Slovenia is a relatively small country considering its size and the number of inhabitants; secondary level hospitals are found in small towns with close-knit communities where protected information may leak quickly. In addition, the protection of personal information privacy became one of the most pressing security concerns for record keepers, which became more challenging after the introduction of the European General Data Protection Regulation (GDPR) in 2014 and the 2007 Personal Data Protection Act in Slovenian legislation [51-53].

Five types of ethical dilemmas were unanimously placed at the lowest end of the priority by Slovenian HCPs: social inequality, involuntary hospitalization, biomedical research, organ transplantation, and vaccination refusal. Regarding the last two, organ transplantation is legally well regulated and performed in only one tertiary center for the whole country; the Institute for transplantation of organs and Tissues of the Republic of Slovenia is a member of the Eurotransplant organization [54].

Despite some tendency among parents to refuse the vaccination of their children, the percentage of vaccinated children nevertheless remains high in Slovenia and therefore this is not of concern for HCPs in secondary and tertiary level institutions, but rather in primary healthcare institutions that do not have ethics committees of their own.

In all our HCP groups, a high percentage (94.2%) of respondents resolved ethical dilemmas in discussion with their colleagues whereas no differences between secondary and tertiary level institutions were found. The second most frequent approach to resolving ethical dilemmas in all three HCP groups was a discussion with the head of department (55.2%); nurses and other HCPs working in the secondary level institutions used this option more fre-

quently compared to their peers working in tertiary level institutions. Medical council meeting is judged as a very important platform for discussing ethical dilemmas among physicians with no differences found between secondary and tertiary level institutions. HECs are consulted more often in tertiary level institutions (35.3%) compared to secondary level institutions, where HECs are consulted counseled very rarely (4.0%). Physicians in secondary level institutions have significantly higher share and odds for discussing their ethical dilemmas within their family circle compared to physicians working in tertiary level institutions (24.0% vs. 5.9%; OR = 5.1) whereas nurses in secondary level institution preferred mediation compared to nurses in tertiary level institutions (5.7% vs. 1.5%; OR = 4.[illegible]ther HCPs in secondary level institutions had higher odds ratio compared to tertiary level institutions for deciding alone (OR = 11). Discussing ethical dilemmas with co-workers proved to be the most frequently chosen strategy by the respondents in our study. Data information from Moeller et al study how HCPs solve ethical dilemmas after they reviewed 100 cases of ethical consultations showed that the reasons for consultations could be divided into one of eight general categories: conflict over withholding treatment, conflict over withdrawing treatment, futility issues, decisional capacity of patient in question, wishes of patient unknown, patient noncompliant with medical regimen, issues with DNR status, and other [55].

In our previous nation-wide study on experiences of intensivists in intensive care units on end-of life attitudes and how to proceed when faced with ethical dilemmas, merely 60% of the study participants (intensivists) knew how to proceed when facing an ethical dilemma and 23% of all the participants had consulted an HEC before. Furthermore, 42% of the respondents knew the name of the head of HEC in their institution, whereas 17% of them reported not to have HEC in their institution [56].

In our study, it would be interesting to see with whom the physicians consult in cases of end-of-life decision making ethical dilemmas. Nevertheless, when facing ethical dilemmas,

the most common way of dealing with them is to consult the colleagues and/or the head of department. In secondary centers, physicians are significantly less likely to consult HECs compared to those working in tertiary level institutions (OR = 0.1). Nurses in secondary level institutions preferred mediation compared to those working in tertiary level institutions (OR = 4). Regarding the use of HECs, no differences were found between all three HCP groups. In the study on Croatian physicians and nurses, HEC was asked by 12% of physicians and by only 3% of nurses [41] which was much lower than in comparable tertiary institutions in Slovenia.

Our results show that among physicians there was the highest share (30.7%) of those who were unaware of standard procedures for solving ethical dilemmas compared to nurses (13.2%) and other HCPs (6.7%). Very high share of physicians (41.3%), nurses (53.3%), and other HCPs (64.0%) were not aware of existing standard procedures for solving ethical dilemmas. Jointly, this results in a very high share of all HCPs that do not know or/and are unaware of standard procedures, despite the fact that one third of physicians compared to 19.7% and 13.6% of nurses and other HCPs, respectively, recalled that there were undiscussed ethical dilemmas, which they thought should have been discussed. This result reflects that of all HCP profiles who were included into the study some of them rarely confront with ethical dilemmas, while few of them confront more frequently. That issue has been previously shown in our study where 60% of intensivists know how to proceed in case of ethical dilemmas and 23% in the past already consulted HECs in secondary and tertiary level institutions [56]. On questions regarding the existence of HECs in their hospitals[illegible].4% of all HCPs answered positively, that is, that they are aware of it. Among the physicians working in the tertiary level institutions 15 (33,3%) consulted with HECs in 2014 However, none of the physicians and of the other HCPs in secondary level institutions consulted with HECs in 2014. The correspond-

ing number for nurses is 12 (6.9%) and 3 (6.4%) for other HCPs. However, we should stress that HEC in Slovenia are still in the process of building up their operational capacity.

Generally, the intended role of HECs is well known (individual case consultations, education of HCPs, and policy formation) [33-35]. In line with that, HCPs in our study, when ranking, agreed that the most important roles of HECs are staff education, review of difficult cases, and development of protocols as well as improving communication. To both of these questions a very high percentage of HCPs in all groups answered that HECs in the hospitals are very important for resolving ethical dilemmas (84.6%) and that regular formal education about ethics is needed (87.6%). Therefore, it is not surprising that with respect to teaching methods in ethics, all HCPs placed regular formal education in university programs as their first choice and learning from senior co-workers and workshop learning as on the second and third strategies, respectively. It is noteworthy that for healthcare ethics consultation a core competencies and standards should be developed [57].

Limitations

. Despite random sampling methods of sample representativeness, we observed underrepresentation of physicians and overrepresentation of nurses which is related to numerically non-adequate responses in HCPs groups; this could be a source of bias in interpretation of our results. The questionnaire was given only to HCPs, but not to the patients or lay persons accompanying or visiting the patients.

Conclusions

In this study, we included all profiles of HCPs working in secondary and tertiary level institutions in Slovenia who are confronted with various ethical dilemmas during daily work.

The sample size was calculated accordingly to the number of all HCPs from all hospitals and with respect to the profiles of all HCPs to ensure our samples are as representative as possible. Besides pointing to well-known ethical dilemmas, our study clearly shows that waiting periods for diagnostics and treatment of the patients and suboptimal performance due to poor interpersonal relationships on the ward are those types of ethical dilemmas that healthcare policy makers and hospital management should be aware of as much as possible. Due to their interdependence, the two ethical dilemmas probably have to be understood and solved mutually, at least in Slovenian healthcare environment. The most important role of HECs is staff education, followed by improving communication, supplementing the hospital guidelines for resolving ethical dilemmas, and review of difficult cases.

Acknowledgements

We thank all the Slovenian health-care providers (physicians, nurses and all others who take care for the benefit of the patients in the hospitals) for their kind participation in the study and the management of hospitals for cooperation and support.

Special thanks to Kristijan Armeni for proofreading, editing, and correcting the final draft.

References

1. Beauchamp TL, Childres JF. The principles of biomedical ethics. 6th ed. New York: Oxford University Press; 2009.
2. Tishchenko P, Yudin B. Bioethics and Journalism. Moscow: Publishing House “Adamant”; 2011.

3. Levine C. The Seattle ‘God Committee’: A Cautionary Tale. Health Affairs Blog [Internet]. 2009 Nov [cited 2017 Aug 1]. Available from: http://healthaffairs.org/blog/2009/11/30/the-seattle-god-committee-a-cautionary-tale/.
4. ten Have H. Global bioethics: an introduction. London, New York: Routledge; 2016.
5. Kälvemark S, Höglund AT, Hansson MG, Westerholm P, Arnetz B. Living with conflicts-ethical dilemmas and moral distress in the health care system. Soc Sci Med. 2004;58(6):1075-84.
6. Epstein EG, Hamric AB. Moral distress, moral residue, and the crescendo effect. J Clin Ethics. 2009;20(4):330-42.
7. Annas GJ. The case of Baby Jane Doe: child abuse or unlawful Federal intervention? Am J Public Health. 1984;74(7):727-9.
8. White M. The End at the Beginning. Ochsner J. 2011;11(4):309-16.
9. National Library of Medicine. The C. Everett Koop papers: Congenital birth defects and the medical rights of children: the “Baby Doe” controversy [Internet]. Bethesda: National Library of Medicine; 2016 [cited 2017 Aug 1]. Available from: http://profiles.nlm.nih.gov/ps/retrieve/Narrative/QQ/p-nid/86/.
10. Fine RL. From Quinlan to Schiavo: medical, ethical, and legal issues in severe brain injury. Proc (Bayl Univ Med Cent). 2005;18(4):303-10.
11. Bremer A, Herrera MJ, Axelsson C, Martí DB, Sandman L, Casali GL. Ethical values in emergency medical services: a pilot study. Nurs Ethics. 2015;22(8):928-42.
12. Schubert M, Clarke SP, Aiken LH, de Geest S. Associations between rationing of nursing care and inpatient mortality in Swiss hospitals. Int J Qual Health Care. 2012;24(3):230-8.

13. DuVal G, Clarridge B, Gensler G, Danis M. A national survey of U.S. internists' experiences with ethical dilemmas and ethics consultation. J Gen Intern Med. 2004;19(3):251-8.
14. Borovečki A, Orešković S, ten Have H. Ethics and the structures of health care in the European countries in transition: hospital ethics committees in Croatia. BMJ. 2005;331(7510):227-9.
15. Steinkamp N, Gordijn B, Borovecki A, Gefenas E, Glasa J, Guerrier M, et al. Regulation of healthcare ethics committees in Europe. Med Health Care Philos. 2007;10(4):461-75.
16. Williamson L, McLean S, Connell J. Clinical ethics committees in the United Kingdom: towards evaluation. Med Law Int. 2007;8(3):221-38.
17. Williamson L. Empirical assessments of clinical ethics services: implications for clinical ethics committees. Clin Ethics. 2007;2(4):187-92.
18. Goldim JR, Raymundo MM, Fernandes MS, Lopes MH, Kipper DJ, Francisconi CF. Clinical Bioethics Committees: a Brazilian experience. J Int Bioethique. 2008;19(1-2):181-92.
19. McGee G, Caplan AL, Spanogle JP, Asch DA. A national study of ethics committees. Am J Bioeth. 2001;1(4):60-4.
20. Slowther AM, McClimans L, Price C. Development of clinical ethics services in the UK: a national survey. J Med Ethics. 2012;38(4):210-4.
21. Marcus BS, Carlson JN, Hegde GG, Shang J, Venkat A. Evaluation of viewpoints of health care professionals on the role of ethics committees and hospitals in the resolution of clinical ethical dilemmas based on practice environment. HEC Forum. 2016;28(1):35-52.

22. Marcus BS, Shank G, Carlson JN, Venkat A. Qualitative analysis of healthcare professionals' viewpoints on the role of ethics committees and hospitals in the resolution of clinical ethical dilemmas. HEC Forum. 2015;27(1):11-34.
23. Czarkowski M, Kaczmarczyk K, Szymańska B. Hospital Ethics Committees in Poland. Sci Eng Ethics. 2015;21(6):1525-35.
24. Stolper M, Molewijk B, Widdershoven G. Learning by doing. Training health care professionals to become facilitator of moral case deliberation. HEC Forum. 2015;27(1): 47-59. doi: 10.1007/s10730-014-9251-7;;
25. Molewijk AC, Abma T, Stolper M, Widdershoven G Teaching ethics in the clinic. The theory and practice of moral case deliberation. J Med Ethics. 2008;34(2):120-4. doi: 10.1136/jme.2006.018580.
26. Carravallah LA, Reynolds LA, and Woolford SJ. Lessons for Physicians from Flint's Water Crisis Laura. AMA Journal of Ethics. 2017;19(10): 1001-10.
27. Declaration of Helsinki. Recommendations guiding physicians in clinical research. Adopted by the World Medical Association in 1964. Wis Med J. 1967;66(1):25-6.
28. Žakelj T, Primožič J. The work of the Republic of Slovenia for National Medical Ethics Committee. In: Parežnik R, Grosek Š, Kremžar B, Muzlovič I, Podbregar M, Gradišek P, editors. School of intensive care: 4th year. Conference publication; Novo mesto, Slovenia. Ljubljana: Slovenian Society for Intensive Care Medicine; 2012. p. 140-4.
29. Milčinski J, Stražiščar S. Teaching medical ethics: Faculty of medicine Ljubljana, Yugoslavia. J Med Ethics. 1980;6(3):145-8.
30. Craig RP, Middleton CL, O'Connell LJ. Ethics committees: a Practical approach. St. Louis: Catholic Health Association; 1986.

31. Borovečki A, Sass HM. The use of checklists in clinical ethics. Special edition for the 9th World Congress in Bioethics of the International Association of Bioethics. Zagreb: Andrija Štampar School of Public Health, School of Medicine, University of Zagreb; 2008.
32. Borovečki A, ten Have H, Orešković S. Ethics committees in Croatian healthcare institutions: the first study about their structure, functions and some reflections on the major issues and problems. HEC Forum. 2006;18(1).49-60.
33. UNESCO. Guide N°.1 Establishing Bioethics Committees. Paris: United Nations Educational, Scientific and Cultural Organization; 2005. 72 p.
34. UNESCO. Guide N°.2 Establishing Bioethics Committees. Paris: United Nations Educational, Scientific and Cultural Organization; 2005. 72 p.
35. UNESCO. Guide N°.3 Establishing Bioethics Committees. Paris: United Nations Educational, Scientific and Cultural Organization; 2005. 78 p.
36. Fox E. Strategies to Improve Health Care Ethics Consultation: Bridging the Knowledge Gap. AMA J Ethics. 2016;18(5):528-33.
37. Kučan R, Grošelj J. Functioning of hospital ethics committees in medical institutions in Slovenia: facing healthcare professionals with ethical dilemmas and with the work of the Medical Ethics Committee of the University Clinical Centre Ljubljana [student research thesis]. Ljubljana: University of Ljubljana; 2015.
38. The R Foundation. The R Project for Statistical Computing. Version 3.1.3 [software]. 2015 Mar 9 [cited 2017 Aug 1]. Available from: https://www.r-project.org/.
39. Anthony R, Artino Jr., Jeffrey S. La Rochelle, Kent J. Dezee & Hunter Gehlbach () Developing questionnaires for educational research: AMEE Guide No. 87, Medical Teacher. 2014; 36(6): 463-74, DOI: 10.3109/0142159X.2014.889814

40. Ivanc B. Constitutional review of the Slovenian health law. European Journal of Health Law. 2007;14 (4):335-47.
41. Sorta-Bilajac I, Baždarič K, Brozovič B, Agich G. Croatian physicians' and nurses' experience with ethical issues in clinical practice. J Med Ethics. 2008;34:450-5.
42. Sorta-Bilajac I, Baždarić K, Žagrović MB, Jančić E, Brozović B, Čengic T, et al. How nurses and physicians face ethical dilemmas – the Croatian experience. Nurs Ethics. 2011;18(3):341-55.
43. Hurst SA, Perrier A, Pegoraro R, Reiter-Theil S, Forde R, Slowther AM, et al. Ethical difficulties in clinical practice: experiences of European physicians. J Med Ethics. 2007;33:51-7.
44. Bagnasco A, Cadorin L, Barisone M, Bressan V, Iemmi M, Prandi M, et al. Ethical dimensions of paediatric nursing: A rapid evidence assessment. Nurs Ethics. 2016. Epub 2016 Mar 22.
45. Barnitt R. Ethical dilemmas in occupational therapy and physical therapy: a survey of practitioners in the UK National Health Service. J Med Ethics. 1998;24(3):193-9.
46. Luz KR, Oliveira Vargas MA, Schmidtt PH, Barlem ELD, Tomaschewski-Barlem JG, Rosa LM. Ethical problems experienced by oncology nurses. Rev Lat Am Enfermagem. 2015;23(6):1187-94.
47. Losa Iglesias ME, Becerro de Bengoa VR, Salvadores Fuentes P. Moral distress related to ethical dilemmas among Spanish podiatrists. J Med Ethics. 2010;36(5):310-4.
48. Ćurković M, Milošević M, Borovečki A, Mustajbegović J. Physicians' interpersonal relationships and professional standing seen through the eyes of the general public in Croatia. Patient Prefer Adherence. 2014;8:1135-42.

49. Aleksandrova S. Survey on the experience in ethical decision-making and attitudes of Pleven University Hopital physicians toward ethics consultation. Med Health Care Philos. 2008;11:35-42.
50. Norberg A, Udén G. Gender differences in moral reasoning among physicians, registered nurses and enrolled nurses engaged in geriatric and surgical care. Nurs Ethics. 1995;2(3):233-42.
51. Neame RLB. Privacy protection for personal health information and shared care records. Inform Prim Care. 2014;21(2):84-91.
52. General Data Protection Regulation 2016 [Internet]. GDPR; 2016 [cited 2017 Aug 1]. Available from: http://www.eugdpr.org/.
53. Public Information Access Act 2003. The Official Journal of the Republic of Slovenia No. 24/2003.
54. The Removal and Transplantation of Human Body Parts for the Purposes of Medical Treatment Act 2000. The Official Journal of the Republic of Slovenia No. 12/2000.
55. Moeller JR, Albanese TH, Garchar K, Aultman JM, Radwany S, Frate D. Functions and out-comes of a clinical medical ethics committee: A review of 100 consults. HEC Forum. 2012;24:99-114.
56. Grošelj U, Oražem M, Kanič M, Vidmar G, Grosek Š. Experiences of Slovene ICU physicians with end-of-life decision making: a nation-wide survey. Med Sci Monit. 2014;20:2007-12.
57. Tarzian AJ. Health care ethics consultation: an update on core competencies and emerging standards from the American Society For Bioethics and Humanities' core competencies update task force. Am J Bioeth. 2013;13(2):3-13.

Supporting information

Author contribution

ŠG, RK, JG, MO, UG, VA, JL, and BI conceived the study protocol; ŠG, RK, JG, and BI participated in the design and coordination of the study; ŠG, RK, and JG collected data; ŠG, RK, JG, and BI supervised data collection; VE provided statistical analysis; ŠG, RK, AB, and BI interpreted the results; ŠG, RK, JG, BI, and AB carried out the literature search; ŠG, RK, AB, and BI drafted the present manuscript; ŠG, RK, AB, and BI revised the manuscript. All authors read and approved the final version of the manuscript.

Consent for publication

The authors have given their full consent for publication.

Funding

The study was funded as part of the research program Terciar—Project development of the Committee for Medical Ethics University Medical Centre Ljubljana and Clinical Ethics (No: 20140215) of the University Medical Centre Ljubljana in the years 2014–2016.

Ethical approval

The study was approved by the National Medical Ethics Committee of the Republic of Slovenia on January 1, 2015 (ref. No. of approval 43/01/15 and ref. No. 0120-68/2018/8) The research presented for re-evaluation is observational, all data of included healthcare (HEC) providers were collected in that way that anonymity of included HEC providers was fully obtained in the way that personal information of the HEC providers at the departments cannot

be recognized. Therefore the NMEC RS stated that for participation in research and use of necessary data to include them no written informed consent is needed.

Prof. Stefan Grosek, MD, PhD.,
Specialist in pediatrics, specialist in intensive medicine
University Medical Centre Ljubljana, Division of Obstetrics and Gynecology

Department of Perinatology, Neonatal intensive Care Unit, Šlajmerjeva 4, 1000 Ljubljana, Slovenia

Divison of Surgical Services, Department of Pediatric surgery and Intensive Therapy, Pediatric Intensive Care Unit
Bohoričeva 20, 1000 Ljubljana, Slovenia

Department of Pediatrics, Medical Faculty, University of Ljubljana

Bohoriceva 20, 1525 Ljubljana

GSM ++ 386 (0)41 684 206

T ++ 386 (0)1 522 60 11

T ++ 386 (0)1 522 62 10

F ++ 386 (0)1 522

E stefan.grosek@mf.uni-lj.si

E stefan.grosek@kclj.si

W www.kclj.si

Subject: PLOS ONE Decision: Revision required [PONE-D-17-33632] - [EMID:5f292b405c782b7e]

Dear Editor and Reviewers

Thank you for your review of the manuscript. We have taken into consideration the concerns and comments as you can see in the attached document. The points raised were considered once again and were more critically discussed in the revised manuscript.

The authors found the reviewers' comments very helpful and are grateful for their efforts in improving the manuscript.
The revised manuscript has been read and approved by all authors. Parts of the manuscript that have been changed in response to concerns of the reviewers are underlined. All minor changes have been corrected, but not underlined. We would be grateful for your critical evaluation.

PONE-D-17-33632
The first nation-wide study on facing and solving ethical dilemmas among healthcare professionals in Slovenia: do hospital ethics committees really matter?
PLOS ONE

We would appreciate receiving your revised manuscript by Feb 18 2018 11:59PM. When you are ready to submit your revision, log on to http://pone.edmgr.com/ and select the 'Submissions Needing Revision' folder to locate your manuscript file.

If you would like to make changes to your financial disclosure, please include your updated statement in your cover letter.

To enhance the reproducibility of your results, we recommend that if applicable you deposit your laboratory protocols in protocols.io, where a protocol can be assigned its own identifier (DOI) such that it can be cited independently in the future. For instructions see: http://journals.plos.org/plosone/s/submission-guidelines#loc-laboratory-protocols

Please include the following items when submitting your revised manuscript:

- A rebuttal letter that responds to each point raised by the academic editor and reviewer(s). This letter should be uploaded as separate file and labeled 'Response to Reviewers'.
- A marked-up copy of your manuscript that highlights changes made to the original version. This file should be uploaded as separate file and labeled 'Revised Manuscript with Track Changes'.
- An unmarked version of your revised paper without tracked changes. This file should be uploaded as separate file and labeled 'Manuscript'.

We look forward to receiving your revised manuscript.

Kind regards,

Ganesh Dangal, MD, FICS
Academic Editor
PLOS ONE

Journal Requirements:

When submitting your revision, we need you to address these additional requirements.

1. Please ensure that your manuscript meets PLOS ONE's style requirements, including those for file naming. The PLOS ONE style templates can be found at http://www.journals.plos.org/plosone/s/file?id=wjVg/PLOSOne_formatting_sample_main_body.pdf and http://www.journals.plos.org/plosone/s/file?id=ba62/PLOSOne_formatting_sample_title_authors_affiliations.pdf

***Reply to Q1**: We checked again and we believe that our manuscript meets PLOS ONE's style requirements.*

2. Thank you for your ethics statement: "The study was approved by the Slovene National Medical Ethics Committee (Decision No.43/01/15) but waived the need for informed consent because the study was purely observational."

Please clarify this statement within the manuscript to indicate whether the Slovene National Medical Ethics Committee waived the informed consent requirement. In addition, please state in the manuscript whether the Slovene National Medical Ethics Committee approved the collection procedure for the lists of public employee names and "the personal information of the employees at the department." Thank you for your attention to these requests.

Reply to Q2*: Thank you for this question and need for clarification. We checked our statements once again and therefore sent an inquiry again to the National Ethics Committe of Republic Slovenia (NEC RS) about the questions raised by the Editor. Their final statement released by March 19, 2018 (ref. No 0120-68/2018/8) was: Begin of quote: »The research presented for reevaluation is observational, all data of included healthcare (HEC) providers were collected in that way that anonimity of included HEC providers was fully obtained in the way that personal information of the HEC providers at the departments cannot be recognized. Therefore the NEC RS stated that for participation in research and use of necessary data to include them no written informent consent is needed«. End of quote.*

3. We suggest you thoroughly copyedit your manuscript for language usage, spelling, and grammar. If you do not know anyone who can help you do this, you may wish to consider employing a professional scientific editing service.

Whilst you may use any professional scientific editing service of your choice, PLOS has partnered with both American Journal Experts (AJE) and Editage to provide discounted services to PLOS authors. Both organizations have experience helping authors meet PLOS guidelines and can provide language editing, translation, manuscript formatting, and figure formatting to ensure your manuscript meets our submission guidelines. To take advantage of our partnership with AJE, visit the AJE website (http://learn.aje.com/plos/) for a 15% discount off AJE services. To take advantage of our partnership with Editage, visit the Editage website (www.editage.com) and enter referral code PLOSEDIT for a 15% discount off Editage services. If the PLOS editorial team finds any language issues in text that either AJE or Editage has edited, the service provider will re-edit the text for free.

Upon resubmission, please provide the following:

- The name of the colleague or the details of the professional service that edited your manuscript
- A copy of your manuscript showing your changes by either highlighting them or using track changes (uploaded as a *supporting information* file)
- A clean copy of the edited manuscript (uploaded as the new *manuscript* file)

Reply to Q3: *Please find the name and address of professional copyedit professional:*

Prevajanje, Sebastijan Armeni, s.p. (translation service, sole ownership)

Malejeva 15, 1110 Ljubljana

Slovenia (8009384000)

(registration nr: 8009384000)

4. We note you have included a table to which you do not refer in the text of your manuscript. Please ensure that you refer to Table 6 in your text; if accepted, production will need this reference to link the reader to the Table.

Reply to Q4*: Thank you for you remark. We checked again and found that that Table 6 refer to text of our manuscript on page 22, lines 346-347.*

5. Review Comments to the Author

Please use the space provided to explain your answers to the questions above. You may also include additional comments for the author, including concerns about dual publication, research ethics, or publication ethics. (Please upload your review as an attachment if it exceeds 20,000 characters)

Reviewer #3: This is an interesting paper reporting for the first time on ethical difficulties encountered by Slovenian health care providers as well as their views regarding the usefuleness of Healthcare Ethics Committees. Recruitment allows the authors to compare doctors, nurses, and other health care professionals, as well as secondary and tertiairy care hospitals. My comments are as follows:

Major comments:

1) The survey was administered anonymously and this seems appropriate. However, was the identity of those who participated or not known within each department?

Reply to Q1: *Thank you for you question. We may confirm that the identity of those who participated or not within each department was known only to the human management administration (HMA) who delivered envelope with the questionnaire to participants. The participants were chosen on random basis from hospital ID numbers and the ID numbers were sent to the HMA who delivered questionnaires. Those who returned questionnaires consented to participate. Their names were anonymous to the team of researchers.*

2) The sort of dilemma being reported here would be better caracterised as a difficulty than as a dilemma. Many are not situations where HCP do not know what to do. Rather, they are situations where there is something morally problematic about the situation. I would urge the authors to correct the language.

Reply to Q2: *We agree with the reviewer that dealing with situations in the hospital are not always such to be presented as moral or ethical dilemmas but rather difficulties. But if the they bring HCP's in moral distress than it can be understand as ethical dilemmas. We were particularly interested of those situations when it bring them into moral distress so we believe that it better represents concerns we rised with the questionnaire. And to continue, in the questionnaire we used this therm » ethical dilemma« therefore, we cannot change it now for the purpose of publication.*

3) There is something strange about the finding that doctors encountered more ethical difficulties than nurses did. I would have welcomed discussion of possible explanations for this.

Reply to Q3: *Thank you to rise this interesting issue which need further research and clarification on this matter.At the moment we cannot explicitly answer to this question. When we discusses ourselves to explain this discrepancy being found we were not able to find good or acceptable answer. What we may say at the moment is that we need certainly to do research on this topics and reveal what it would be the cause and what lies behind theses results.*

One of possible answers could be that differences between Slovenian and European nurses are due to different role and competencies of Slovenian nurses compared to other nurses in mostly the Northern part of Europe. Slovenian nurses have much less competencies recognized during clinical work which bring them to be in more subordinate role (lower autonomy). They strictly follow physicinas orders because they through education process competencies of more important clinical decisions are not allowed to be performed by them. We strongly believe that in the future therir competencies will increase.

4) The authors should be careful about their interpretation of the frequency of different types of difficulties. For example, organ transplantation and involuntary hospitalization may simply be more rare. That they are infrequently problematic may not mean that they are not problematic, simply that they happen less frequently.

Reply to Q4: *Thank you for this valuable comments. We fully agree with your comments that we should be careful about the interpretation of the frequency of different types of dilemmas or difficulties. Our primary aim was to include as many as possible relevant ethical dilemmas or difficulties raised by HCPs not only in the hospitals but also in social media (TV, facebook, twitter, etc..) encountered not only by HCPs but also by lay persons. All of these issues you raised, i.e. organ transplantation, involuntary hospitalization and other would need new special research related only to these topics.*

But, overall our results clearly showed that doctors were consistently encountered more ethical issues compared to nurses and other HCPs.

5) Regarding the usefuless of HECs reported by HPCs: did the responses of those who had consulted HECs differ from those of professionals who had not done so, and if so how?

Reply to Q5: *Thank you for questioning this interesting issue. We also tested if differences exists and we found that those HCPs who consulted HECs compared to those who did not consult HECs indeed encountered more frequent dilemmas related end-of-life treatment withdrawal (p=0.002), suboptimal performance due to interpersonal relationship on the ward (p<0.001), unresponsiveness to adverse events in the medical care of patients (p=0.009), and distribution of limited resources (p=0.018).*

Thirty-one out of 443 HCPs who answered the question consulted HECs. Of these, 15 (48.4%), physicians, 12 nurses (38.7%) and 4 (12.9%) other HCPs. Relevant profile data among HCPs who did not consult HECs were 55 (13.3%), 284 (68.9%), and 73 (17.7%). The difference in proportions is statistically significant (p <0.001). Healthcare professionals who consulted with HECs have more frequent dilemmas related end-of-life treatment withdrawal (p = 0.002), compared with those HCPs who did not consult HECs, suboptimal performance due to interpersonal relationship on the ward (p <0.001) unresponsiveness to adverse events in the medical care of patients (p = 0.009 and distribution of limited resources (p = 0.018).

Minor comments:
6) The background of HEC establishment in Slovenia could be more precise. The statement that HECs were established in the 90s, for example, seems in contradiction with the earlier statement that the first was established in the 60s. What happened in fact? Moreover, were HECs under communist rule the same sort of entities as afterwards? Please say more.

Reply to Q6: *Thank you to elucidate to us our inconsistency in writing about the establishement of HECs in Slovenia. We corrected the sentence in the manuscript: »In Slovenia the first Ethics committee was established at the Faculty of Medicine, University of Ljubljana, in the second half of 1960's after the Helsinki declaration on human research came into effect (26). We change also the reference 24 with prof. Trontelj's reference on this issue.*

To answer you second part of the question whether HECs under communist rule the same sort of entities as afterwards we may answer that despite communist rule ethical issues and concerns were not different before or after communist rule. Despite some ethicists were communists all of them shared high ethical personality not influence by communists.

7) On page 3, the authors state that "It was among the first in Europe and its primary role was to evaluate ethical adequacy of doctoral research projects." Were these project PhD research by graduate students? I suspect what is intended here is "medical research projects".

Reply to Q7: *We agree with the reviewer's comments and we accordingly correct the sentence.*

Reviewer #4: Thank You for giving me the opportunity to read Your work! Please see the attached document with comments inserted in the manuscript!

OVERALL COMMENTS:

Thank You for giving me the chance to read this interesting work! You will find my comments in the document. The main concerns that I have with the manuscript are:

Reply to reviewer's #4 comment: *First of all we would like to thank you for reviewing our work which you positively commented as interesting work, which we honestly believe truly is, too. It gave us answers to many issues related to ethical dilemmas among Slovene HCPs and other HCPs which were not yet studied in Slovenia. We will will answer to all your concerns item by item.*

Subtitle: very appealing but not able to answer in this study.

2) Background –lack of important information for the reader to understand the results. Lack of a wider perspective on ethics support.

3) Methods - absence of easily follow the scientific process

4) Questionnaire - Major concerns about the validation process and how the items were selected (both ethical dilemmas and HECs role) as well as misleading writing about unaware/aware.

5) Research questions – to broad and not all are answered.

6) Results – has to be adjusted according to chosen research questions-

7) Discussion – please elaborate more on the discussion and reflect on the results. As it is read now the discussion is a bit shallow and repeats the results more than discussing the underlying reason and meaning of it.

8) Conclusion – develop what conclusion You are drawing out of these results? What does the results mean?

TITLE:

Page 1; line 2-3: To who/what? Appealing title! But, would You say that this could be answered with data from the study specific questionnaire?

Reply to reviewer's #4 comment: *It is true very appaealing and it is also true that in the way of presentations and interpretation of our results we were not able to answer. Therefore we will remove this subtitle from the manuscript title.*

ABSTRACT:

Page 2; line 36: 2 missing?

Reply to reviewer's #4 comment: *We apologize for this mistake. The correct number is 485 and was corrected in the manuscript.*

Page 2; lines 42-44: Why only mention how doctors (physicians I suppose, or do all of them have a PhD?) solve the ethical issues? What about nurses? The research question is: "…how do HCPs solve them"?

Reply to reviewer's #4 comment: *Thank you for this comment. Indeed, it is true that physicians, nurses and othe HCPs commonly discuss ethical dilemmas with co-workers and with head of department. Please find correction in the Abstract section.*

INTRODUCTION:

Page 3; lines 53-54: What are the ethical challenges? Since the context of the study is very general it would be good to add examples of ethical issues from previous research. There are sometimes major differences related to type of care. For example paediatrics vs elderly care.

Reply to reviewer's #4 comment: *Yes indeed different ethical challenges arouse through last few decades many of them also because of immense technical innovations in medicine along with broad social movements. As an intensivist of almost forty years of clinical work in intensive care unit, me together with my colleagues were able to face with all these ethical challenges which were not only related to advancement in medicine but also to shortage of money, interpersonnel disputes, and finally also to dissolution of communism and formerYugoslavia in independent new country, like Slovenia, and others. I was able to work with adult and neonatal and pediatric specialists in different fields of clinical medicine and during discussion we found these questions prepared for questionnaire those which we are facing daily.*

We wanted to get a broader and bigger picture of ethical challenges HCPs face daily in Slovene hospitals at secondary and tertiary levels. And we believed that we were able to collect important data on ethical dilemmas/difficulties which affect us as a HCPs and also lay persons daily in Slovenia. Moreover, at this moment we found that some of ethical challenges

we brought out are the most important in Slovenia while others are also important but at the moment to less extent perturbating Slovene health system.

In conclusion, recently we published three papers which deal with special type of care, end-of-life decisions, and attitudes in all Slovenian intensive care units adult and pediatric which gave us pretty much information how important is studying and evaluating different aspects of ethical dilemmas.

Please see references below:

A Cross-sectional Study Among Healthcare and Non-healthcare Students in Slovenia and Croatia About Do-not Resuscitate Decision-making.
Puc J, Obadić P, Erčulj V, Borovečki A, Grosek Š.
Zdr Varst. 2019 Jun 26;58(3):139-147. doi: 10.2478/sjph-2019-0018. eCollection 2019 Sep.

Notes on the Development of the Slovenian Ethical Recommendations for Decision-Making on Treatment and Palliative Care of Patients at the End of Life in Intensive Care Medicine.
***Grosek S**, Orazem M, Groselj U.*
Pediatr Crit Care Med. 2018 Aug;19(8S Suppl 2):S48-S52. doi: 10.1097/PCC.0000000000001606. Review.

End-of-life decisions in 34 Slovene Intensive Care Units: a nationwide prospective clinical study.
*Orazem M, Groselj U, Stojan M, Majdic N, Vidmar G, **Grosek S**.*
Minerva Anestesiol. 2017 Jul;83(7):728-736. doi: 10.23736/S0375-9393.17.11711-6. Epub 2017 Feb 15.

Attitudes of Slovene paediatricians to end-of-life care.
***Grosek S**, Orazem M, Kanic M, Vidmar G, Groselj U.*
J Paediatr Child Health. 2016 Mar;52(3):278-83. doi: 10.1111/jpc.13006. Epub 2015 Oct 29.

Experiences of Slovene ICU physicians with end-of-life decision making: a nation-wide survey.
*Groselj U, Orazem M, Kanic M, Vidmar G, **Grosek S**.*
Med Sci Monit. 2014 Oct 21;20:2007-12. doi: 10.12659/MSM.891029

GROSEK, Štefan (avtor, urednik), GROŠELJ, Urh (avtor, urednik), ORAŽEM, Miha (avtor, urednik), BOROVŠAK, Zvonko, EBERT MOLTARA, Maja, GRADIŠEK, Primož, IVANC, Blaž, KAPŠ, Rafael, KOSEC, Lučka, KRIŽNAR, Tomaž, KRŽIŠNIK-ZORMAN, Simona, LOPUH, Mateja, JEREB, Matjaž, PETKOVŠEK, Robert, PLETERSKI-RIGLER, Dušica, RADŠEL, Peter, SINKOVIČ, Andreja, SOK, Mihael, STRAHOVNIK, Vojko, ŠVIGELJ, Viktor, VLAHOVIĆ, Dušan, VOGA, Gorazd, ŽAKELJ, Tone. Ethical recommendations for decision-making on treatment and palliative care of patients at the end of life in intensive care

medicine : a joint statement of the Slovenian Society of Intensive Medicine and the Slovenian National Medical Ethics Committee. 1st ed. Ljubljana: University Medical Centre, 2016. 56 str. ISBN 978-961-6442-68-8. [COBISS.SI-ID 282205184]

Page 3; line64: ref.

Reply to reviewer's #4 comment: *References 7-10 were added.*

Page 3; lines 67-68: What about Individuals working with ethics in institutions that does not have a HECs? They are trained in guiding ethics within the team and it can have the same effect as when a case is sent to a HEC. When ethical issues are dealt with within the team the relational knowledge of the case is guaranteed. I think this section would be improved if this is further problematized. There is a lot of research done on HCPs, specialized in ethics, having a great impact on recognizing, improving, and resolving diverse ethical dilemmas.

Reply to reviewer's #4 comment: *We cannot more than to agree with the reviewer's comments. Indeed there is a lot of research done on HCPs, specialized in ethics. But such possibilities are especially recognized mostly in Northern parts of Europe where through training HCPs to become facilitator of moral case deliberation became important partners in solving ethical dilemmas. (HEC Forum. 2015 Mar;27(1):47-59. doi: 10.1007/s10730-014-9251-7; Learning by doing. Training health care professionals to become facilitator of moral case deliberation.Stolper M[1], Molewijk B, Widdershoven G; ; J Med Ethics. 2008 Feb;34(2):120-4. doi: 10.1136/jme.2006.018580.; Teaching ethics in the clinic. The theory and practice of moral case deliberation.Molewijk AC[1], Abma T, Stolper M, Widdershoven G). In Slovenia resolving ethical dilemmas is still in hands of the HECs and no training is available at the moment for individual training.*

So ve added next sentence and references in the part of the manuscript: » Not only HECs but also other possibilities of resolving ethical dilemmas developed mostly in the Northern parts of Europe where through professional training of HCPs they become facilitators of moral case deliberation among co-workers with whom they work every day. (ref. HEC Forum. 2015 Mar;27(1):47-59. doi: 10.1007/s10730-014-9251-7; Learning by doing. Training health care professionals to become facilitator of moral case deliberation.Stolper M[1], Molewijk B, Widdershoven G; ; J Med Ethics. 2008 Feb;34(2):120-4. doi: 10.1136/jme.2006.018580.; Teaching ethics in the clinic. The theory and practice of moral case deliberation.Molewijk AC[1], Abma T, Stolper M, Widdershoven G).

In Slovenia, resolving ethical dilemmas is still in hands of the HECs and no official ethics training is available at the moment for HECs. Even in some difficult cases other countries, i.e. with well established HECs recognized that physicians should be more active when the physicians are faced with situations when people may be affected due to their ignorance. (AMA Journal of Ethics® October 2017, Volume 19, Number 10: 1001-1010 MEDICAL EDUCATION

Lessons for Physicians from Flint's Water Crisis Laura A. Carravallah, MD, Lawrence A. Reynolds, MD, and Susan J. Woolford, MD, MPH).

Page 4; lines 75-76: Due to?

Reply to reviewer's #4 comment: *Thank you for your short notice. Why HECs were introduced so late cannot be rationally explained. Slovenia despite communistic rule was widely opened to the Western part of Europe, got influences from there but it seems that nobody recognized that ethics should have played important part in everyday clinical work.*

Page 4: line 86: This is very interesting, because (as mentioned above) ethics support can be performed in other ways as well. For example: ethics consults or within the inter-professional team. However when HEC are present at hospitals one could argue for that it creates expectations of ethics support and reflections on ethical issues. I think, for the "background" up to here, it is too narrow in terms of ethics support. Even if the objectives are to study HECs, the reader would benefit from knowing that there are other ways of conducting ethics support and a lot of research is done related to that.

Reply to reviewer's #4 comment: *Thank you for your valuable comments. You raised the same questions above where we tried to answer on its. We all agree that this question should be deleted because it may have negative impacts on those who tried to build competent HECs through many decades. However new ways of resolving the ethical problems aroused in the very last years through teaching ethics of HCPs who became facilitators and was already presented above and also inserted in the manuscript.*

Page 4; line 87-88: Please clarify? Does ethical dilemmas being perceived differently if HECs is presence?

Reply to reviewer's #4 comment:*The correct and honest answer to this question is that we don't know. We can only asume that without HECs ethical dilemmas were not perceived differently compare to being presented. Nevertheless the structure of resolving ethical dilemmas in Slovenia now is that we have HECs with aim of developing ethical perspectives in daily clinical work, and resolving different ethical dilemmas presented by the questionnaire in the study.*

Therefore, we replaced these sentence with a new one: "A motivation for this study to evaluate how HCP face with ethical dilemmas and how they resolve it."

METHODS:

Page 5; line 102: The design section would benefit from more distinctly describing the overall design with the study.

The entire methodology of the study should then be systematically described and logically motivated in relation to purpose as well as anchored in relevant methodical literature. As it is now, sampling/participants is for example described in the design. It would be better if the Methods section was divided into: **Overall Design, Study Context and Participants, Data Collection (including the questionnaire), Data Analyses –Statistics, a separate heading of Ethical Considerations is missing for example**: *"The study has been approved by…….Written information about the purpose of the study and ……was given to each HCP….."*

Page 5; lines118-119: Ethical Considerations

Reply to reviewer's #4 comment: *Please find rearrangement and amendment of the section Methods as suggested by the reviewer.*

RESULTS:

Page 9; line 122: Study specific? Please add if so.

Reply to reviewer's #4 comment: *Yes, correction done.*

Page 5 and 6; lines 123-129: Please consider merging this into the next paragraph for easier reading.

Reply to reviewer's #4 comment: *Please find merging this part as you suggested.*

Page 6; line 133: How do you motivate this section? Do You assume that interpersonal relations is a part of solving ethical issues? Could these questions have had an impact on the coming answers by first asking about interpersonal relations and then about how they solve ethical dilemmas. Was there a risk that the informants' thoughts ruled against relationships? I think this can be addressed in the Limitation paragraph.

Reply to reviewer's #4 comment: *Thank you for this question. When we looked back again if the sentence is correctly written we found a mistake which was then interpreted in the way as you did.*

The correct sentence should be as follows: "In the second part, we aimed at determining how often are HCPs faced with ethical dilemmas and to estimate how do they encounter ethical dilemmas in the domains listed in the questionnaire in their professional work."

After changing the sentence you further questions are not any more an issue to be dissused.

Page 6; lines135-138: Why did You integrate questions about ethics training? I think it leads to non-stringent materials. For now, data is already collected, but I think You should consider concentrating on:

1) How often are HCPs faced with ethical dilemmas?

2) How they solved the recognized ethical dilemmas

3) What percentage of HCPs are aware of the existence of the HEC and how well it is integrated into everyday clinical practice?

Reply to reviewer's #4 comment: *We thank for the reviewer's suggestion. In the manuscript we shall concentrate on these 3 main topics as were suggested by the reviewer.*

Save the estimation of interpersonal relations at their workplace and the best method for education on medical ethics to an other publication.

Reply to reviewer's #4 comment: *We thank the reviewer's comment, therfore we removed this part from the sentence as well as also the estimation of interpersonal relations in other parts of the manuscript.*

Page 6; line 135-138: Please clarify how this could leave knowledge about if HECs really matters?

Reply to reviewer's #4 comment: *We thank the reviewer for this valuable comment. We remove the Subtitle.*

Page 6; lines 140: The question: *"3.2. PLEASE CHOOSE FROM THE LIST BELOW **THREE MOST IMPORTANT** AREAS OF RESPONSIBILITY OF THE MEDICAL ETHICS COMMITTEE"* is not described in the method section. The fact that respondents have to choose the three most important roles most likely have an impact on the results. This must be described in a transparent way.

Reply to reviewer's #4 comment: *We thank for this comment. In the manuscript we amended the sentence: "We were interested in learning what percentage of HCPs are aware of the existence of the HEC and what are the most important area of responsibility of the HECs.*

Page 6, lines 140: The validation process of the questionnaire must be much more clear. What about, test-retest, cognitive interviews, psychometric tests etc. Did You follow well established guidelines. References?

Reply to reviewer's #4 comment: *Thank you for your comments.* Seven-step approach to questionnaire development as recommended by AMEE guidelines were followed (Anthony R. Artino Jr., Jeffrey S. La Rochelle, Kent J. Dezee & Hunter Gehlbach (2014) Developing questionnaires for educational research: AMEE Guide No. 87, Medical Teacher, 36:6, 463-474, DOI: 10.3109/0142159X.2014.889814):

Step 1: Literature review

Step 2: Interviews / focus groups

Step 3: Synthesize the literature review and interviews

Step 4: Develop items

Step 5: Expert validation (=face validity)

Step 6: Cognitive interviews (skupaj s pilot testingom)

Step 7: Pilot testing.

After first four steps the last three steps were followed with final testing reliability:

5. *Establishing **face validity**: different experts (physicians, members of CMEs, lawyers and lay persons) evaluated the content of the questionnaire. Questionnaire was changed accordingly. It was checked further for wording, possible double-barrelled, confusing and misleading questions by two psychometricians.*

6. ***Pilot test**: the pilot questionnaire was given and answered by 35 health-care professionals of different occupational profiles. Participants commented the questionnaire, pointed possible difficulties they had with understanding or answering certain questions. On the basis of comments and results of the statistical analysis, some of the items were dropped from the final questionnaire.*

7. ***Validity:** Exploratory factor analysis on the whole Slovene sample showed all the items measuring frequency of ethical dilemmas loaded substantially (loadings > 0.50) on the same factor. The validity of the questionnaire was assessed further by investigating correlations between general question on ethical dilemma frequency and questions on each specific dilemma frequency. Correlation coefficients varied between 0.27 and 0.52 and were all statistically significant ($p < 0.001$).*

***Reliability**: the questionnaire exhibits high internal consistency (Cronbach α = 0.93). Test-retest reliability was not assessed to ensure/guarantee complete respondent anonymity. The later was crucial for the validity of the obtained results.*

Commented [SG1]: From Vanja Erčulj
7 step approach to questionnaire development as recommended by AMEE guidelines were followed (Anthony R. Artino Jr., Jeffrey S. La Rochelle, Kent J. Dezee & Hunter Gehlbach (2014) Developing questionnaires for educational research: AMEE Guide No. 87, Medical Teacher, 36:6, 463-474, DOI: 10.3109/0142159X.2014.889814):
Step 1: Literature review
Step 2: Interviews / focus groups
Step 3: Synthesize the literature review and interviews
Step 4: Develop items
Step 5: Expert validation (=face validity)
Step 6: Cognitive interviews (skupaj s pilot testingom)
Step 7: Pilot testing.

Prve 4 korake je naredil dr Ivanc že s kvalitativno raziskavo, in sicer intervjuji s predsedniki KME. Poleg priporočenih korakov smo ugotavljali še psihometrične lastnosti, in sicer veljavnost merjenja z eksploratorno faktorsko analizo in zanesljivost merjenja z izračunom Cronbachovega koeficienta alfe

Page 7; lines 153: What were the inclusion criteria? Please add.

Reply to reviewer's #4 comment: *We include in the study HCPs (physicians, nurses), and other HCPs, (laboratory technicians and engineers, radiologic engineers, clinical psychologists, nurse assistants, technicians and enginers of biochemistry, pharmacists, social workers, physiotherapists, respiratory therapists, speech therapists, hygiene technicians, psychologists).*

Please find participants who were included under section Study Context and Participants.

Please find inclusion criteria in the manuscript.

Page 7; lines 160: Repetition.

Reply to reviewer's #4 comment: *To be deleted*

Page 8; line177: Dichotomised? Ref?

Reply to reviewer's #4 comment: *Correction was made.*

Page 8; lines 191-192: The range is quite large (26-85%). This could interestingly be discussed.

Reply to reviewer's #4 comment: *We appreciate your comment, but we cannot explain why the range is large. We approached to each hospitals the same but from the beginning we felt that in some hospitals the questionnaire research was not accepted as we expected.*

Page 9; Table 1: Religion: I am curious why this sociodemographic question was asked, do you think it will have an impact on the results? There is no data in the result section corresponding to the question. Please, deliberate if this should be excluded.

Reply to reviewer's #4 comment: *Thank you for your valuable comment. Yes, we believe that this sociodemographic question may have an impact on the results. Spring et al showed that religious affiliation has an impact on end.of-life decisions in European intensive care units.(ref.* The importance of religious affiliation and culture on end-of-life decisions in European intensive care units. Sprung CL, Maia P, Bulow HH, Ricou B, Armaganidis A, Baras M, Wennberg E, Reinhart K, Cohen SL, Fries DR, Nakos G, Thijs LG; **Ethicus Study** Group. Intensive Care Med. 2007 Oct;33(10):1732-9. Epub 2007 Jun 1. Erratum in: Intensive Care Med. 2007 Oct;33(10):1859).

Also in our study published recently (ref. A Cross-sectional Study Among Healthcare and Non-healthcare Students in Slovenia and Croatia About Do-not Resuscitate Decision-making. Puc J, Obadić P, Erčulj V, Borovečki A, Grosek Š. Zdr Varst. 2019 *Jun 26;58(3):139-147. doi: 10.2478/sjph-2019-0018. eCollection 2019 Sep.) we found that gender, religious beliefs, and type of study are important factors associated with the decisions pertaining to the respect for autonomy, patient's best interest, and initiation or withholding of resuscitation*).

Other HCP: Please specify what kind of professions this is. Social workers, nurse assistants?

Reply to reviewer's #4 comment: *In the Legend of Table 1 we added the list of other HCPs, (laboratory technicians and enginers, radiologic enginers, clinical psychologists, nurse assistants, technicians and enginers of biochemistry, pharmacists, social workers,*

physiotherapists, respiratory therapists, speech therapists, hygiene technicians, psychologists).

Page 10; line 218: It is not clear in the manuscript how and why the predetermined ethical dilemmas were chosen in the questionnaire? Based on previous research? Please, clarify! If it is not based on research the scientific value must be discussed.

Reply to reviewer's #4 comment: *Our predetermined ethical dilemmas chosen in the questionnaire were based on our previous student research work in the biggest tertiary hospital in Slovenia in 2015. In this research we tested and validated what are the most important ethical dilemmas. Please see below a reference:*

(ref. KUČAN, Rok, GROŠELJ, Jon. Delovanje področnih komisij za medicinsko etiko v zdravstvenih zavodih v Sloveniji : soočanje zdravstvenih delavcev z etičnimi dilemami in z delovanjem komisije za medicinsko etiko Univerzitetnega kliničnega centra Ljubljana. Ljubljana: [T. Šket], 2015. VI, 49 f., graf. prikazi, tabele. [COBISS.SI-ID 2800044])

Page 13, line 239: Disagreement with who? Does this differ between professions? Nurses vs physicians, Nurses vs nurses, nurses vs others? "An individual" appears as to general. The results would be more interesting if the professions were not clustered.

Reply to reviewer's #4 comment:

Thank you for your comment. Indeed, we were interested in broader defintion of disagreement as you cited in your comment. We know that differences exist between different professions, but our aim was exactly what we asked for to get general information on disagreement with an individuals's professional work among HCPs, whether between physicians, nurses, oter HCPs or physicians-nurses, and so on. Namely, from the literature we know that one of the hallmarks of an unhealthy work environment is poor communication between doctors and nurses, i.e. disagreement (ref. Pediatrics. 2017 Sep;140(3). pii: e20170670. doi: 10.1542/peds.2017-0670. Epub 2017 Aug 16. Physician-Nurse Interactions in Critical Care. Khowaja-Punjwani S, Smardo C, Hendricks MR, Lantos JD). Overall, nearly 20% of nurses leave their first job within a year of being hired (ref. Kovner CT, Brewer CS, Fatehi F, Jun J. What does nurse turnover rate mean and what is the rate? Policy Polit Nurs Pract. 2014;15(3–4):64–71). Poor communication leads to medical errors (Aiken LH, Clarke SP, Sloane DM, Lake ET, Cheney T. Effects of hospital care environment on patient mortality and nurse outcomes. J Nurs Adm. 2008;38(5):223–229).

Page 13; line 245: Being a HCP and researcher from one of the Scandinavian countries this is a bit hard to understand. Please shortly describe the differences in context in the methods-section under "Study Context and Participants".

Reply to reviewer's #4 comment:

Thank you for your valuable comment. Please find in the section under ”Study Context and Participants” short description:

“Due to different professional and academic workload and obligations of HCPs and other HCPs between secondary and tertiary level institutions we expect that different ethical dilemmas will be perceived within each groups and subgroups of HCPs and other HCPs.

Page 15; lines 264-265: Another reason for writing about other ways of dealing with ethical dilemmas then HECs. From previous research I have learnt that there is a great value of shared inter-professional ethics case reflections.

Reply to reviewer’s #4 comment:

We agree with the reviewer’s comment.

Page 16, Table3: Reaction? Please clarify what is intended.

Suggestion: Association between types of HCPs and ways of dealing with ethical dilemmas.

Reply to reviewer’s #4 comment:

Thank you for your suggestion, we gladly accepted it and replaced it.

Page 16, Table3: This comes for the second time and this time with different numbers? Please correct

Reply to reviewer’s #4 comment:

Correction was made. Corrected item: Contact the media

Page 18; line 284: What is a family circle? The patients family?

Reply to reviewer’s #4 comment:

Family circle means HCPs’ family. Please find correction in the manuscript

Page 19; Fig2: Please see above!

Reply to reviewer’s #4 comment:

Correction was made. Corrected title is: Fig 2. Ways of dealing to ethical dilemmas…

Page 19; lines 299: What are the standard procedures? If this should be written here it must be described in the background. Standard procedures could differ between countries, cities, hospitals, units etc.

Reply to reviewer's #4 comment:

Thank you for this comment. The question to the participants was whether the HCPs are aware of standard procedures for solving ethical dilemmas. Under hospital regulations' acts, each Slovenian hospital should have a document how to resolve indivividual conflicts whether between HCPs or HCPs and patients. Moreover each hospital should also have a person who is dealing with such conflicts, i.e. ombudsman of patient rights, complaint board, as well as Hospital Ethics Committee. In the UMC Ljubljana safety officers for patients, employees, visitors and others are employed.

Whenever somebody perceives professional deviation, whether ethical or nonethical , an institution should have ombudsam, committee or safety officers to whom anybody can addreess complaints.

Page 19; line 300: Yes of being unaware, or being aware? Please clarify!

Reply to reviewer's #4 comment:

The correct answer is: yes of being aware

Page 19; Table 4: Aware or unaware of? (yes: aware; no: unaware)

Reply to reviewer's #4 comment:

Please find corrected version in the manuscript and table:

Yes of being aware

No of being unaware

Page 20; line 320: Usually, sentences do not start with numbers. Please check writing guidelines.

Reply to reviewer's #4 comment:

Correction was made in the manuscript: More than one third (36.5%) of all…

Page 23; Table 6: Please specify from were these roles were collected. Are there a common service or role description for the 14 hospitals? How is HECs practice performed

Reply to reviewer's #4 comment:

Thank you for your comment. Our predetermined ethical dilemmas chosen in the questionnaire were based on our previous student research research work in the biggest tertiary hospital in Slovenia in 2015. In this research we tested and validated what are the most important ethical dilemmas. Please see below a reference:

(ref. KUČAN, Rok, GROŠELJ, Jon. Delovanje področnih komisij za medicinsko etiko v zdravstvenih zavodih v Sloveniji : soočanje zdravstvenih delavcev z etičnimi dilemami in z delovanjem komisije za medicinsko etiko Univerzitetnega kliničnega centra Ljubljana. Ljubljana: [T. Šket], 2015. VI, 49 f., graf. prikazi, tabele. [COBISS.SI-ID 2800044])

DISCUSSION:

Page 25; lines 355-377: Physicians? A nurse can be a doctor to when earned a PhD.

Reply to reviewer's #4 comment:

We agree with the reviewer's comment. Please find changes in the entire manuscript.

Please see above, other needs to be defined.

Reply to reviewer's #4 comment:

Corrections have been made in the manuscript with definitions of otherHCPs.

This needs to be explained or removed, "the nature" is on a further philosophical level.

Reply to reviewer's #4 comment:

Correction has been made. "the nature" was removed.

Please clarify, maybe misunderstanding due to language?

Reply to reviewer's #4 comment:

Please find corrected text: ...and what is the way of dealing resolving ethical dilemmas. This study reveals several important findings.

This is really interesting, a lot of other studies confirm the opposite. What are the underlying interpretation of the phenomenon?

Reply to reviewer's #4 comment:

Thank you for your comment. We really don't know the cause and the right answer.In postsocialistic health system in Slovenia physicians are under great pressure to serve all health expectations from patients "offered" them by health politics while limited resources are available. The lack of physicians and modern technology for diagnostics make physicians dissatisfied and under moral distress when they cannot fulfill the expectations of the patients.

Correct English?

Comparable with the 20 predefined ethical dilemmas in the present study? Or just defined as ethical dilemmas and not specified.

Reply to reviewer's #4 comment:

This is correct statement.The "various" means defined as ethical dilemmas and not specified.

Almost 15 year old reference.

Reply to reviewer's #4 comment:

We believe that this reference is still valid for the purpose of discussion.

Page 26; line391-393: Which indeed is a great argument for educating physicians but also nurses and other HCPs

Reply to reviewer's #4 comment:

We can no more than to agree.

How is this related to the results in the present study? Please problematize!

Reply to reviewer's #4 comment:

This results concur with ours where we found that 35.3% od physicians working in the tertiary level hospitals would discuss with hospital ethical committee compared to only 4% of them in secondary level hospitals when facing with ethical dilemma (p=0.015). No differences were found for nurses and other HCPs. Therefore we can draw a conclusion that not only physicians in the secondary level hopsitals but also nurses and other HCPs in secondary and teriary level hospitals are probably not enough aware of presence and therefore using hospital ethics committees when facing with ethical dilemmas. More education is probably needed as well spreading of information among HCPs in the hospitals that HECs are available and ready to help in solving ethical dilemmas.

Page 26; lines400: Very nice discussion

Reply to reviewer's #4 comment: *for this comment.*

Page 27; line 417-418: What is the interpretation of this? Are they as close to the patient and the suffering as the physicians and nurses? Do they see the patient in another way?

Reply to reviewer's #4 comment:

The answer cannot be interpreted unanimously. What we can derive from this answer is that we should conduct a new research in which we would take questions on this issue raised. One of the possible answers would be a.) that the otherHCPs have access to large amount of data, information whent they take care of the patients specifically to their profession and that seems to them the most important, b.) that nobody teach them how to manage and interpret many personal information they came to their ears.

Page 28; line448-449: ref?

Reply to reviewer's #4 comment:

No reference is available

.Page 29; lines 466-467: Please clarify as the statement is hard to understand? What do you mean with "…are available only when considering the reasons for consultations with HECs" ?

Reply to reviewer's #4 comment:

Thank you for your comment. Please find corrected text: "Data information from Moeller et al study how HCPs solve ethical dilemmas after they reviewed 100 cases of……..".

Page 30; lines483-484: Repetition, please correct.

Reply to reviewer's #4 comment:

Correction made! Please find that we replaced "nurses" with "to those"

Page 30; lines 502-504: Doesn´t this first say that none of the other HCPS consulted the HECs and then in the second sentence that 6.4% did it anyway?

Reply to reviewer's #4 comment:

Please find my correction. We added additional sentence for clarification: "Among the physicians working in the tertiary level institutions 15 (33,3%) consulted with HECs in 2014".

Page 31; lines 502-504: According to my opinion: Just because no one uses the services does not mean that it does not matter. The fundamental work of the HECs seems to need a review and efforts have to be made to inform the HCPs about the support that the HEC can offer.

Reply to reviewer's #4 comment:

We agree with the reviewer's opinion and we removed this sentence.

Page 31; lines 511-513: Isn´t this a circle argument? The most important role is in concordance with the most important role? Please clarify!

Reply to reviewer's #4 comment:

We agree with the reviewer's opinion and we removed this sentence.

LIMITATIONS:

Page 31; lines 523-527: However, do You believe that it is possible to explore the same research question in such different contexts`? The ethical dilemmas are varying leading to different needs of ethics support. According to literature ethics is not context dependent but still ethics support needs to be tailored based on the experiences perceived by the HCPs working in that specific care institution. Imagine the difference between hospitals for mental diseases and intensive care units in regards to for example the time limit the dilemma has to be resolved within. Enabling scientific stringency my perception is that support services should be studied in the same type of care and cultural affiliation.

Reply to reviewer's #4 comment:

Thank you fo your valubl comments. We removed thi sentence form the text.

Page 32; lines 531-532: This could be future research

Reply to reviewer's #4 comment:

Agree with the reviewer's comment. We reoved this sentence and leave it for future research

ACKNOWLEGEMENTSch!

Page 32; lines 546-547: This is results, what conclusion are You drawing out of these results? What does it mean?

Reply to reviewer's #4 comment:

Thank you for the reviewer's comment.

CONCLUSIONS:

Page 32; lines 550: As authors? This can be read as You only are thanking physicians that answered the questionnaire. I think it is polite to be thankful to all the participants for contributing with their experiences by completing the questionnaire. Consider adding.

Reply to reviewer's #4 comment:

We corrected acknowledgment. Please find it in the manuscript. "We thank all the Slovenian health-care providers (physicians, nurses and all others who take care for the benefit of the patients in the hospitals) for their kind participation in the study and the ~~Slovene Society of Intensive Medicine~~ management of hospitals for cooperation and support. "

ACKNOWLEDGEMENTS:

Page 32; lines 550-551:

As authors? This can be read as You only are thanking physicians that answered the questionnaire. I think it is polite to be thankful to all the participants for contributing with their experiences by completing the questionnaire. Consider adding.

Reply to reviewer's comment: *We agree with the reviewer's comment, however this was type writing error and was corrected accordingly. Please find corrected sentence: "We thank all the Slovenian health-care providers (physicians, nurses and all others who take care for the benefit of the patients in the hospitals) for their kind participation in the study and the ~~Slovene Society of Intensive Medicine~~ management of hospitals for cooperation and support. "*
