# Supplementary material for: The first nationwide study on facing and solving ethical dilemmas among healthcare professionals in Slovenia
Source: PLoS One. 2020 Jul 14;15(7):e0235509. doi: 10.1371/journal.pone.0235509 (PMC7360038; doi:10.1371/journal.pone.0235509)
Supplement: Attachment — Submitted filename: Grosek et al RESPONSE to REVIEWERS-R1.asd.docx [file pone.0235509.s006.docx]

# **Prof. Stefan Grosek, MD, PhD.,**

# **Specialist in pediatrics, specialist in intensive medicine**

University Medical Centre Ljubljana, Division of Obstetrics and Gynecology

Department of Perinatology, Neonatal intensive Care Unit, Šlajmerjeva 4, 1000 Ljubljana, Slovenia

Divison of Surgical Services, Department of Pediatric surgery and Intensive Therapy, Pediatric Intensive Care Unit Bohoričeva 20, 1000 Ljubljana, Slovenia

Department of Pediatrics, Medical Faculty, University of Ljubljana

 Bohoriceva 20, 1525 Ljubljana

**GSM ++ 386 (**0)41 684 206

**T** **++ 386 (**0)1 522 60 11

**T** **++ 386 (**0)1 522 62 10

**F** **++ 386 (**0)1 522

**E** [stefan.grosek@mf.uni-lj.si](mailto:stefan.grosek@mf.uni-lj.si)

**E** [stefan.grosek@kclj.si](mailto:stefan.grosek@kclj.si)

**W** [www.kclj.si](http://www.kclj.si)

**Subject:** PLOS ONE Decision: Revision required [PONE-D-17-33632] - [EMID:5f292b405c782b7e]

Dear Editor and Reviewers

Thank you for your review of the manuscript. We have taken into consideration the concerns and comments as you can see in the attached document. The points raised were considered once again and were more critically discussed in the revised manuscript.

The authors found the reviewers’ comments very helpful and are grateful for their efforts in improving the manuscript.

The revised manuscript has been read and approved by all authors. Parts of the manuscript that have been changed in response to concerns of the reviewers are underlined. All minor changes have been corrected, but not underlined. We would be grateful for your critical evaluation.

PONE-D-17-33632
The first nation-wide study on facing and solving ethical dilemmas among healthcare professionals in Slovenia: do hospital ethics committees really matter?
PLOS ONE

We would appreciate receiving your revised manuscript by Feb 18 2018 11:59PM. When you are ready to submit your revision, log on to <http://pone.edmgr.com/> and select the 'Submissions Needing Revision' folder to locate your manuscript file.

If you would like to make changes to your financial disclosure, please include your updated statement in your cover letter.

To enhance the reproducibility of your results, we recommend that if applicable you deposit your laboratory protocols in protocols.io, where a protocol can be assigned its own identifier (DOI) such that it can be cited independently in the future. For instructions see: <http://journals.plos.org/plosone/s/submission-guidelines#loc-laboratory-protocols>

Please include the following items when submitting your revised manuscript:

- A rebuttal letter that responds to each point raised by the academic editor and reviewer(s). This letter should be uploaded as separate file and labeled 'Response to Reviewers'.
- A marked-up copy of your manuscript that highlights changes made to the original version. This file should be uploaded as separate file and labeled 'Revised Manuscript with Track Changes'.
- An unmarked version of your revised paper without tracked changes. This file should be uploaded as separate file and labeled 'Manuscript'.

We look forward to receiving your revised manuscript.

Kind regards,

Ganesh Dangal, MD, FICS
Academic Editor
PLOS ONE

**Journal Requirements:**

When submitting your revision, we need you to address these additional requirements.
 
1. Please ensure that your manuscript meets PLOS ONE's style requirements, including those for file naming. The PLOS ONE style templates can be found at
<http://www.journals.plos.org/plosone/s/file?id=wjVg/PLOSOne_formatting_sample_main_body.pdf> and <http://www.journals.plos.org/plosone/s/file?id=ba62/PLOSOne_formatting_sample_title_authors_affiliations.pdf>

***Reply to Q1****: We checked again and we believe that our manuscript meets PLOS ONE's style requirements.*

2. Thank you for your ethics statement: "The study was approved by the Slovene National Medical Ethics Committee (Decision No.43/01/15) but waived the need for informed consent because the study was purely observational."

Please clarify this statement within the manuscript to indicate whether the Slovene National Medical Ethics Committee waived the informed consent requirement. In addition, please state in the manuscript whether the Slovene National Medical Ethics Committee approved the collection procedure for the lists of public employee names and "the personal information of the employees at the department." Thank you for your attention to these requests.

***Reply to Q2****: Thank you for this question and need for clarification. We checked our statements once again and therefore sent an inquiry again to the National Ethics Committe of Republic Slovenia (NEC RS) about the questions raised by the Editor. Their final statement released by March 19, 2018 (ref. No 0120-68/2018/8) was: Begin of quote: »The research presented for reevaluation is observational, all data of included healthcare (HEC) providers were collected in that way that anonimity of included HEC providers was fully obtained in the way that personal information of the HEC providers at the departments cannot be recognized. Therefore the NEC RS stated that for participation in research and use of necessary data to include them no written informent consent is needed«. End of quote.*

3. We suggest you thoroughly copyedit your manuscript for language usage, spelling, and grammar. If you do not know anyone who can help you do this, you may wish to consider employing a professional scientific editing service.  

Whilst you may use any professional scientific editing service of your choice, PLOS has partnered with both American Journal Experts (AJE) and Editage to provide discounted services to PLOS authors. Both organizations have experience helping authors meet PLOS guidelines and can provide language editing, translation, manuscript formatting, and figure formatting to ensure your manuscript meets our submission guidelines. To take advantage of our partnership with AJE, visit the AJE website (<http://learn.aje.com/plos/>) for a 15% discount off AJE services. To take advantage of our partnership with Editage, visit the Editage website ([www.editage.com](http://www.editage.com)) and enter referral code PLOSEDIT for a 15% discount off Editage services.  If the PLOS editorial team finds any language issues in text that either AJE or Editage has edited, the service provider will re-edit the text for free.
 
Upon resubmission, please provide the following:

- The name of the colleague or the details of the professional service that edited your manuscript
- A copy of your manuscript showing your changes by either highlighting them or using track changes (uploaded as a *supporting information* file)
- A clean copy of the edited manuscript (uploaded as the new *manuscript* file)

***Reply to Q3:*** *Please find the name and address of professional copyedit professional:*

***Prevajanje, Sebastijan Armeni, s.p. (translation service, sole ownership)***

***Malejeva 15, 1110 Ljubljana***

***Slovenia (8009384000)***

***(registration nr: 8009384000)***

4. We note you have included a table to which you do not refer in the text of your manuscript. Please ensure that you refer to Table 6 in your text; if accepted, production will need this reference to link the reader to the Table.

***Reply to Q4****:* *Thank you for you remark. We checked again and found that that Table 6 refer to text of our manuscript on page 22, lines 346-347.*

**5. Review Comments to the Author**

Please use the space provided to explain your answers to the questions above. You may also include additional comments for the author, including concerns about dual publication, research ethics, or publication ethics. (Please upload your review as an attachment if it exceeds 20,000 characters)

**Reviewer #3:** This is an interesting paper reporting for the first time on ethical difficulties encountered by Slovenian health care providers as well as their views regarding the usefuleness of Healthcare Ethics Committees. Recruitment allows the authors to compare doctors, nurses, and other health care professionals, as well as secondary and tertiairy care hospitals. My comments are as follows:

Major comments:

1) The survey was administered anonymously and this seems appropriate. However, was the identity of those who participated or not known within each department?

***Reply to Q1:*** *Thank you for you question. We may confirm that the identity of those who participated or not within each department was known only to the human management administration (HMA) who delivered envelope with the questionnaire to participants. The participants were chosen on random basis from hospital ID numbers and the ID numbers were sent to the HMA who delivered questionnaires. Those who returned questionnaires consented to participate. Their names were anonymous to the team of researchers.*

2) The sort of dilemma being reported here would be better caracterised as a difficulty than as a dilemma. Many are not situations where HCP do not know what to do. Rather, they are situations where there is something morally problematic about the situation. I would urge the authors to correct the language.

***Reply to Q2:*** *We agree with the reviewer that dealing with situations in the hospital are not always such to be presented as moral or ethical dilemmas but rather difficulties. But if the they bring HCP's in moral distress than it can be understand as ethical dilemmas. We were particularly interested of those situations when it bring them into moral distress so we believe that it better represents concerns we rised with the questionnaire. And to continue, in the questionnaire we used this therm » ethical dilemma« therefore, we cannot change it now for the purpose of publication.*

3) There is something strange about the finding that doctors encountered more ethical difficulties than nurses did. I would have welcomed discussion of possible explanations for this.

***Reply to Q3:*** *Thank you to rise this interesting issue which need further research and clarification on this matter.At the moment we cannot explicitly answer to this question. When we discusses ourselves to explain this discrepancy being found we were not able to find good or acceptable answer. What we may say at the moment is that we need certainly to do research on this topics and reveal what it would be the cause and what lies behind theses results.*

*One of possible answers could be that differences between Slovenian and European nurses are due to different role and competencies of Slovenian nurses compared to other nurses in mostly the Northern part of Europe. Slovenian nurses have much less competencies recognized during clinical work which bring them to be in more subordinate role (lower autonomy). They strictly follow physicinas orders because they through education process competencies of more important clinical decisions are not allowed to be performed by them. We strongly believe that in the future therir competencies will increase.*

4) The authors should be careful about their interpretation of the frequency of different types of difficulties. For example, organ transplantation and involuntary hospitalization may simply be more rare. That they are infrequently problematic may not mean that they are not problematic, simply that they happen less frequently.

***Reply to Q4:*** *Thank you for this valuable comments. We fully agree with your comments that we should be careful about the interpretation of the frequency of different types of dilemmas or difficulties. Our primary aim was to include as many as possible relevant ethical dilemmas or difficulties raised by HCPs not only in the hospitals but also in social media (TV, facebook, twitter, etc..) encountered not only by HCPs but also by lay persons. All of these issues you raised, i.e. organ transplantation, involuntary hospitalization and other would need new special research related only to these topics.*

*But, overall our results clearly showed that doctors were consistently encountered more ethical issues compared to nurses and other HCPs.*

5) Regarding the usefuless of HECs reported by HPCs: did the responses of those who had consulted HECs differ from those of professionals who had not done so, and if so how?

***Reply to Q5:*** *Thank you for questioning this interesting issue. We also tested if differences exists and we found that those HCPs who consulted HECs compared to those who did not consult HECs indeed encountered more frequent dilemmas related end-of-life treatment withdrawal (p=0.002), suboptimal performance due to interpersonal relationship on the ward (p<0.001), unresponsiveness to adverse events in the medical care of patients (p=0.009), and distribution of limited resources (p=0.018).*

*Thirty-one out of 443 HCPs who answered the question consulted HECs. Of these, 15 (48.4%), physicians, 12 nurses (38.7%) and 4 (12.9%) other HCPs. Relevant profile data among HCPs who did not consult HECs were 55 (13.3%), 284 (68.9%), and 73 (17.7%). The difference in proportions is statistically significant (p <0.001). Healthcare professionals who consulted with HECs have more frequent dilemmas related end-of-life treatment withdrawal (p = 0.002), compared with those HCPs who did not consult HECs, suboptimal performance due to interpersonal relationship on the ward (p <0.001) unresponsiveness to adverse events in the medical care of patients (p = 0.009 and distribution of limited resources (p = 0.018).*

**Minor comments:**
6) The background of HEC establishment in Slovenia could be more precise. The statement that HECs were established in the 90s, for example, seems in contradiction with the earlier statement that the first was established in the 60s. What happened in fact? Moreover, were HECs under communist rule the same sort of entities as afterwards? Please say more.

***Reply to Q6:*** *Thank you to elucidate to us our inconsistency in writing about the establishement of HECs in Slovenia. We corrected the sentence in the manuscript: »**In Slovenia the first Ethics committee was established at the Faculty of Medicine, University of Ljubljana, in the second half of 1960's after the Helsinki declaration on human research came into effect (26). We change also the reference 24 with prof. Trontelj's reference on this issue.*

*To answer you second part of the question whether HECs under communist rule the same sort of entities as afterwards we may answer that despite communist rule ethical issues and concerns were not different before or after communist rule. Despite some ethicists were communists all of them shared high ethical personality not influence by communists.*

7) On page 3, the authors state that "It was among the first in Europe and its primary role was to evaluate ethical adequacy of doctoral research projects." Were these project PhD research by graduate students? I suspect what is intended here is "medical research projects".

***Reply to Q7:*** *We agree with the reviewer's comments and we accordingly correct the sentence.*

**Reviewer #4:** Thank You for giving me the opportunity to read Your work! Please see the attached document with comments inserted in the manuscript!

**OVERALL COMMENTS:**

Thank You for giving me the chance to read this interesting work! You will find my comments in the document. The main concerns that I have with the manuscript are:

***Reply to reviewer’s #4 comment:*** *First of all we would like to* ***t****hank you for reviewing our work which you positively commented as interesting work, which we honestly believe truly is, too. It gave us answers to many issues related to ethical dilemmas among Slovene HCPs and other HCPs which were not yet studied in Slovenia. We will will answer to all your concerns item by item.*

Subtitle: very appealing but not able to answer in this study.

2) Background –lack of important information for the reader to understand the results. Lack of a wider perspective on ethics support.

3) Methods - absence of easily follow the scientific process

4) Questionnaire - Major concerns about the validation process and how the items were selected (both ethical dilemmas and HECs role) as well as misleading writing about unaware/aware.

5) Research questions – to broad and not all are answered.

6) Results – has to be adjusted according to chosen research questions-

7) Discussion – please elaborate more on the discussion and reflect on the results. As it is read now the discussion is a bit shallow and repeats the results more than discussing the underlying reason and meaning of it.

8) Conclusion – develop what conclusion You are drawing out of these results? What does the results mean?

**TITLE:**

Page 1; line 2-3: To who/what? Appealing title! But, would You say that this could be answered with data from the study specific questionnaire?

***Reply to reviewer’s #4 comment:*** *It is true very appaealing and it is also true that in the way of presentations and interpretation of our results we were not able to answer. Therefore we will remove this subtitle from the manuscript title.*

**ABSTRACT:**

Page 2; line 36: 2 missing?

***Reply to reviewer’s #4 comment:*** *We apologize for this mistake. The correct number is 485 and was corrected in the manuscript.*

Page 2; lines 42-44: Why only mention how doctors (physicians I suppose, or do all of them have a PhD?) solve the ethical issues? What about nurses? The research question is: “…how do HCPs solve them”?

***Reply to reviewer’s #4 comment:*** *Thank you for this comment. Indeed, it is true that physicians, nurses and othe HCPs commonly discuss ethical dilemmas with co-workers and with head of department. Please find correction in the Abstract section.*

INTRODUCTION:

Page 3; lines 53-54: What are the ethical challenges? Since the context of the study is very general it would be good to add examples of ethical issues from previous research. There are sometimes major differences related to type of care. For example paediatrics vs elderly care.

***Reply to reviewer’s #4 comment:*** *Yes indeed different ethical challenges arouse through last few decades many of them also because of immense technical innovations in medicine along with broad social movements. As an intensivist of almost forty years of clinical work in intensive care unit, me together with my colleagues were able to face with all these ethical challenges which were not only related to advancement in medicine but also to shortage of money, interpersonnel disputes, and finally also to dissolution of communism and formerYugoslavia in independent new country, like Slovenia, and others. I was able to work with adult and neonatal and pediatric specialists in different fields of clinical medicine and during discussion we found these questions prepared for questionnaire those which we are facing daily.*

*We wanted to get a broader and bigger picture of ethical challenges HCPs face daily in Slovene hospitals at secondary and tertiary levels. And we believed that we were able to collect important data on ethical dilemmas/difficulties which affect us as a HCPs and also lay persons daily in Slovenia. Moreover, at this moment we found that some of ethical challenges we brought out are the most important in Slovenia while others are also important but at the moment to less extent perturbating Slovene health system.*

*In conclusion, recently we published three papers which deal with special type of care, end-of-life decisions, and attitudes in all Slovenian intensive care units adult and pediatric which gave us pretty much information how important is studying and evaluating different aspects of ethical dilemmas.*

*Please see references below:*

[*A Cross-sectional Study Among Healthcare and Non-healthcare Students in Slovenia and Croatia About Do-not Resuscitate Decision-making.*](https://www.ncbi.nlm.nih.gov/pubmed/31275441)

*Puc J, Obadić P, Erčulj V, Borovečki A, Grosek Š.*

*Zdr Varst. 2019 Jun 26;58(3):139-147. doi: 10.2478/sjph-2019-0018. eCollection 2019 Sep.*

[*Notes on the Development of the Slovenian Ethical Recommendations for Decision-Making on Treatment and Palliative Care of Patients at the End of Life in Intensive Care Medicine.*](https://www.ncbi.nlm.nih.gov/pubmed/30080807)

***Grosek S****, Orazem M, Groselj U.*

*Pediatr Crit Care Med. 2018 Aug;19(8S Suppl 2):S48-S52. doi: 10.1097/PCC.0000000000001606. Review.*

[*End-of-life decisions in 34 Slovene Intensive Care Units: a nationwide prospective clinical study.*](https://www.ncbi.nlm.nih.gov/pubmed/28206733)

*Orazem M, Groselj U, Stojan M, Majdic N, Vidmar G,* ***Grosek S****.*

*Minerva Anestesiol. 2017 Jul;83(7):728-736. doi: 10.23736/S0375-9393.17.11711-6. Epub 2017 Feb 15.*

[*Attitudes of Slovene paediatricians to end-of-life care.*](https://www.ncbi.nlm.nih.gov/pubmed/26515146)

***Grosek S****, Orazem M, Kanic M, Vidmar G, Groselj U.*

*J Paediatr Child Health. 2016 Mar;52(3):278-83. doi: 10.1111/jpc.13006. Epub 2015 Oct 29.*

[*Experiences of Slovene ICU physicians with end-of-life decision making: a nation-wide survey.*](https://www.ncbi.nlm.nih.gov/pubmed/25335864)

*Groselj U, Orazem M, Kanic M, Vidmar G,* ***Grosek S****.*

*Med Sci Monit. 2014 Oct 21;20:2007-12. doi: 10.12659/MSM.891029*

*GROSEK, Štefan (avtor, urednik), GROŠELJ, Urh (avtor, urednik), ORAŽEM, Miha (avtor, urednik), BOROVŠAK, Zvonko, EBERT MOLTARA, Maja, GRADIŠEK, Primož, IVANC, Blaž, KAPŠ, Rafael, KOSEC, Lučka, KRIŽNAR, Tomaž, KRŽIŠNIK-ZORMAN, Simona, LOPUH, Mateja, JEREB, Matjaž, PETKOVŠEK, Robert, PLETERSKI-RIGLER, Dušica, RADŠEL, Peter, SINKOVIČ, Andreja, SOK, Mihael, STRAHOVNIK, Vojko, ŠVIGELJ, Viktor, VLAHOVIĆ, Dušan, VOGA, Gorazd, ŽAKELJ, Tone. Ethical recommendations for decision-making on treatment and palliative care of patients at the end of life in intensive care medicine : a joint statement of the Slovenian Society of Intensive Medicine and the Slovenian National Medical Ethics Committee. 1st ed. Ljubljana: University Medical Centre, 2016. 56 str. ISBN 978-961-6442-68-8. [COBISS.SI-ID* [*282205184*](https://plus.si.cobiss.net/opac7/bib/282205184?lang=si)*]*

Page 3; line64: ref.

***Reply to reviewer’s #4 comment:*** *References 7-10 were added.*

Page 3; lines 67-68: What about Individuals working with ethics in institutions that does not have a HECs? They are trained in guiding ethics within the team and it can have the same effect as when a case is sent to a HEC. When ethical issues are dealt with within the team the relational knowledge of the case is guaranteed. I think this section would be improved if this is further problematized. There is a lot of research done on HCPs, specialized in ethics, having a great impact on recognizing, improving, and resolving diverse ethical dilemmas.

***Reply to reviewer’s #4 comment:*** *We cannot more than to agree with the reviewer’s comments. Indeed there is a lot of research done on HCPs, specialized in ethics. But such possibilities are especially recognized mostly in Northern parts of Europe where through training HCPs to become facilitator of moral case deliberation became important partners in solving ethical dilemmas. (*[*HEC Forum.*](https://www.ncbi.nlm.nih.gov/pubmed/25218568) *2015 Mar;27(1):47-59. doi: 10.1007/s10730-014-9251-7; Learning by doing. Training health care professionals to become facilitator of moral case deliberation.Stolper M^1^, Molewijk B, Widdershoven G; ;* [*J Med Ethics.*](https://www.ncbi.nlm.nih.gov/pubmed/18234952) *2008 Feb;34(2):120-4. doi: 10.1136/jme.2006.018580.; Teaching ethics in the clinic. The theory and practice of moral case deliberation.Molewijk AC^1^, Abma T, Stolper M, Widdershoven G). In Slovenia resolving ethical dilemmas is still in hands of the HECs and no training is available at the moment for individual training.*

*So ve added next sentence and references in the part of the manuscript:* *» Not only HECs but also other possibilities of resolving ethical dilemmas developed mostly in the Northern parts of Europe where through professional training of HCPs they become facilitators of moral case deliberation among co-workers with whom they work every day. ( ref. [HEC Forum.](https://www.ncbi.nlm.nih.gov/pubmed/25218568) 2015 Mar;27(1):47-59. doi: 10.1007/s10730-014-9251-7; Learning by doing. Training health care professionals to become facilitator of moral case deliberation.Stolper M^1^, Molewijk B, Widdershoven G; ;* [*J Med Ethics.*](https://www.ncbi.nlm.nih.gov/pubmed/18234952) *2008 Feb;34(2):120-4. doi: 10.1136/jme.2006.018580.; Teaching ethics in the clinic. The theory and practice of moral case deliberation.Molewijk AC^1^, Abma T, Stolper M, Widdershoven G).*

*In Slovenia, resolving ethical dilemmas is still in hands of the HECs and no official ethics training is available at the moment for HECs. Even in some difficult cases other countries, i.e. with well established HECs recognized that physicians should be more active when the physicians are faced with situations when people may be affected due to their ignorance. (AMA Journal of Ethics® October 2017, Volume 19, Number 10: 1001-1010 MEDICAL EDUCATION Lessons for Physicians from Flint’s Water Crisis Laura A. Carravallah, MD, Lawrence A. Reynolds, MD, and Susan J. Woolford, MD, MPH).*

Page 4; lines 75-76: Due to?

***Reply to reviewer’s #4 comment:*** *Thank you for your short notice. Why HECs were introduced so late cannot be rationally explained. Slovenia despite communistic rule was widely opened to the Western part of Europe, got influences from there but it seems that nobody recognized that ethics should have played important part in everyday clinical work.*

Page 4: line 86: This is very interesting, because (as mentioned above) ethics support can be performed in other ways as well. For example: ethics consults or within the inter-professional team. However when HEC are present at hospitals one could argue for that it creates expectations of ethics support and reflections on ethical issues. I think, for the “background” up to here, it is too narrow in terms of ethics support. Even if the objectives are to study HECs, the reader would benefit from knowing that there are other ways of conducting ethics support and a lot of research is done related to that.

***Reply to reviewer’s #4 comment:*** *Thank you for your valuable comments. You raised the same questions above where we tried to answer on its. We all agree that this question should be deleted because it may have negative impacts on those who tried to build competent HECs through many decades. However new ways of resolving the ethical problems aroused in the very last years through teaching ethics of HCPs who became facilitators and was already presented above and also inserted in the manuscript.*

Page 4; line 87-88: Please clarify? Does ethical dilemmas being perceived differently if HECs is presence?

***Reply to reviewer’s #4 comment:****The* ***c****orrect and honest answer to this question is that we don’t know. We can only asume that without HECs ethical dilemmas were not perceived differently compare to being presented. Nevertheless the structure of resolving ethical dilemmas in Slovenia now is that we have HECs with aim of developing ethical perspectives in daily clinical work, and resolving different ethical dilemmas presented by the questionnaire in the study.*

*Therefore, we replaced these sentence with a new one:* *“A motivation for this study to evaluate how HCP face with ethical dilemmas and how they resolve it.”*

**METHODS:**

Page 5; line 102: The design section would benefit from more distinctly describing the overall design with the study.

The entire methodology of the study should then be systematically described and logically motivated in relation to purpose as well as anchored in relevant methodical literature. As it is now, sampling/participants is for example described in the design. It would be better if the Methods section was divided into: **Overall Design, Study Context and Participants, Data Collection (including the questionnaire), Data Analyses –Statistics, a separate heading of Ethical Considerations is missing for example***: “The study has been approved by…….Written information about the purpose of the study and ……was given to each HCP…..”*

Page 5; lines118-119: Ethical Considerations

***Reply to reviewer’s #4 comment:*** *Please find rearrangement and amendment of the section Methods as suggested by the reviewer.*

RESULTS:

Page 9; line 122: Study specific? Please add if so.

***Reply to reviewer’s #4 comment:*** *Yes, correction done.*

Page 5 and 6; lines 123-129: Please consider merging this into the next paragraph for easier reading.

***Reply to reviewer’s #4 comment:*** *Please find merging this part as you suggested.*

Page 6; line 133: How do you motivate this section? Do You assume that interpersonal relations is a part of solving ethical issues? Could these questions have had an impact on the coming answers by first asking about interpersonal relations and then about how they solve ethical dilemmas. Was there a risk that the informants' thoughts ruled against relationships? I think this can be addressed in the Limitation paragraph.

***Reply to reviewer’s #4 comment:*** *Thank you for this question. When we looked back again if the sentence is correctly written we found a mistake which was then interpreted in the way as you did.*

*The correct sentence should be as follows*: *“**In the second part, we aimed at determining how often are HCPs faced with ethical dilemmas* *and to estimate how do they encounter ethical dilemmas in the domains listed in the questionnaire in their professional work.”*

*After changing the sentence you further questions are not any more an issue to be dissused.*

Page 6; lines135-138: Why did You integrate questions about ethics training? I think it leads to non-stringent materials. For now, data is already collected, but I think You should consider concentrating on:

1) How often are HCPs faced with ethical dilemmas?

2) How they solved the recognized ethical dilemmas

3) What percentage of HCPs are aware of the existence of the HEC and how well it is integrated into everyday clinical practice?

***Reply to reviewer’s #4 comment:*** *We thank for the reviewer’s suggestion. In the manuscript we shall concentrate on these 3 main topics as were suggested by the reviewer.*

Save the estimation of interpersonal relations at their workplace and the best method for education on medical ethics to an other publication.

***Reply to reviewer’s #4 comment:*** *We thank the reviewer’s comment, therfore we removed this part from the sentence as well as also the estimation of interpersonal relations in other parts of the manuscript.*

Page 6; line 135-138: Please clarify how this could leave knowledge about if HECs really matters?

***Reply to reviewer’s #4 comment:*** *We thank the reviewer for this valuable comment. We remove the Subtitle.*

Page 6; lines 140: The question: *“3.2.	PLEASE CHOOSE FROM THE LIST BELOW* ***THREE MOST IMPORTANT*** *AREAS OF RESPONSIBILITY OF THE MEDICAL ETHICS COMMITTEE”* is not described in the method section. The fact that respondents have to choose the three most important roles most likely have an impact on the results. This must be described in a transparent way.

***Reply to reviewer’s #4 comment:*** *We thank for this comment. In the manuscript we amended the sentence: “We were interested in learning what percentage of HCPs are aware of the existence of the HEC and* *what are the most important area of responsibility of the HECs.*

Page 6, lines 140: The validation process of the questionnaire must be much more clear. What about, test-retest, cognitive interviews, psychometric tests etc. Did You follow well established guidelines. References?

***Reply to reviewer’s #4 comment:*** *Thank you for your comments.* Seven-step approach to questionnaire development as recommended by AMEE guidelines were followed (Anthony R. Artino Jr., Jeffrey S. La Rochelle, Kent J. Dezee & Hunter Gehlbach (2014) Developing questionnaires for educational research: AMEE Guide No. 87, Medical Teacher, 36:6, 463-474, DOI: [10.3109/0142159X.2014.889814](https://doi.org/10.3109/0142159X.2014.889814)):

Step 1: Literature review

Step 2: Interviews / focus groups

Step 3: Synthesize the literature review and interviews

Step 4: Develop items

Step 5: Expert validation (=face validity)

Step 6: Cognitive interviews (skupaj s pilot testingom)

Step 7: Pilot testing.

*After first four steps the last three steps were followed with final testing reliability:*

5. *Establishing* ***face validity****: different experts (physicians, members of CMEs, lawyers and lay persons) evaluated the content of the questionnaire. Questionnaire was changed accordingly. It was checked further for wording, possible double-barrelled, confusing and misleading questions by two psychometricians.*
6. ***Pilot test****: the pilot questionnaire was given and answered by 35 health-care professionals of different occupational profiles. Participants commented the questionnaire, pointed possible difficulties they had with understanding or answering certain questions.  On the basis of comments and results of the statistical analysis, some of the items were dropped from the final questionnaire.*
7. ***Validity:*** *Exploratory factor analysis on the whole Slovene sample showed all the items measuring frequency of ethical dilemmas loaded substantially (loadings > 0.50) on the same factor. The validity of the questionnaire was assessed further by investigating correlations between general question on ethical dilemma frequency and questions on each specific dilemma frequency. Correlation coefficients varied between 0.27 and 0.52 and were all statistically significant (p < 0.001).*

***Reliability****: the questionnaire exhibits high internal consistency (Cronbach α = 0.93). Test-retest reliability was not assessed to ensure/guarantee complete respondent anonymity. The later was  crucial for the validity of the obtained results.*

Page 7; lines 153: What were the inclusion criteria? Please add.

***Reply to reviewer’s #4 comment:*** *We include in the study* *HCPs (physicians, nurses), and other HCPs**, (laboratory technicians and enginers, radiologic enginers, clinical psychologists, nurse assistants, technicians and enginers of biochemistry, pharmacists, social workers, physiotherapists, respiratory therapists, speech therapists, hygiene technicians, psychologists).*

*Please find participants who were included under section Study Context and Participants.*

Please find inclusion criteria in the manuscript.

Page 7; lines 160: Repetition.

***Reply to reviewer’s #4 comment:*** *To be deleted*

Page 8; line177: Dichotomised? Ref?

***Reply to reviewer’s #4 comment:*** *Correction was made.*

Page 8; lines 191-192: The range is quite large (26-85%). This could interestingly be discussed.

***Reply to reviewer’s #4 comment:*** *We appreciate your comment, but we cannot explain why the range is large. We approached to each hospitals the same but from the beginning we felt that in some hospitals the questionnaire research was not accepted as we expected.*

Page 9; Table 1: Religion: I am curious why this sociodemographic question was asked, do you think it will have an impact on the results? There is no data in the result section corresponding to the question. Please, deliberate if this should be excluded.

***Reply to reviewer’s #4 comment:*** *Thank you for your valuable comment. Yes, we believe that this sociodemographic question may have an impact on the results. Spring et al showed that religious affiliation has an impact on end.of-life decisions in European intensive care units.(ref.* [The importance of religious affiliation and culture on end-of-life decisions in European intensive care units.](https://www.ncbi.nlm.nih.gov/pubmed/17541550) Sprung CL, Maia P, Bulow HH, Ricou B, Armaganidis A, Baras M, Wennberg E, Reinhart K, Cohen SL, Fries DR, Nakos G, Thijs LG; **Ethicus** **Study** Group. Intensive Care Med. 2007 Oct;33(10):1732-9. Epub 2007 Jun 1. Erratum in: Intensive Care Med. 2007 Oct;33(10):1859).

Also in our study published recently (ref. [A Cross-sectional Study Among Healthcare and Non-healthcare Students in Slovenia and Croatia About Do-not Resuscitate Decision-making.](https://www.ncbi.nlm.nih.gov/pubmed/31275441)

Puc J, Obadić P, Erčulj V, Borovečki A, Grosek Š. Zdr Varst. 2019 *Jun 26;58(3):139-147. doi: 10.2478/sjph-2019-0018. eCollection 2019 Sep.) we found that gender, religious beliefs, and type of study are important factors associated with the decisions pertaining to the respect for autonomy, patient's best interest, and initiation or withholding of resuscitation*).

Other HCP: Please specify what kind of professions this is. Social workers, nurse assistants?

***Reply to reviewer’s #4 comment:*** *In the Legend of Table 1 we added the list of other HCPs, (laboratory technicians and enginers, radiologic enginers, clinical psychologists, nurse assistants, technicians and enginers of biochemistry, pharmacists, social workers, physiotherapists, respiratory therapists, speech therapists, hygiene technicians, psychologists).*

Page 10; line 218: It is not clear in the manuscript how and why the predetermined ethical dilemmas were chosen in the questionnaire? Based on previous research? Please, clarify! If it is not based on research the scientific value must be discussed.

***Reply to reviewer’s #4 comment:*** *Our* *predetermined ethical dilemmas chosen in the questionnaire were based on our previous student research work in the biggest tertiary hospital in Slovenia in 2015. In this research we tested and validated what are the most important ethical dilemmas. Please see below a reference:*

*(ref. KUČAN, Rok, GROŠELJ, Jon. Delovanje področnih komisij za medicinsko etiko v zdravstvenih zavodih v Sloveniji : soočanje zdravstvenih delavcev z etičnimi dilemami in z delovanjem komisije za medicinsko etiko Univerzitetnega kliničnega centra Ljubljana. Ljubljana: [T. Šket], 2015. VI, 49 f., graf. prikazi, tabele. [COBISS.SI-ID* [*2800044*](https://plus.si.cobiss.net/opac7/bib/2800044?lang=si)*])*

Page 13, line 239: Disagreement with who? Does this differ between professions? Nurses vs physicians, Nurses vs nurses, nurses vs others? “An individual” appears as to general. The results would be more interesting if the professions were not clustered.

***Reply to reviewer’s #4 comment:***

*Thank you for your comment.* *Indeed, we were interested in broader defintion of disagreement as you cited in your comment. We know that differences exist between different professions, but our aim was exactly what we asked for to get general information on disagreement with an individuals's professional work among HCPs, whether between physicians, nurses, oter HCPs or physicians-nurses, and so on. Namely, from the literature we know that one of the hallmarks of an unhealthy work environment is poor communication between doctors and nurses, i.e. disagreement (ref. [Pediatrics.](https://www.ncbi.nlm.nih.gov/pubmed/28814577) 2017 Sep;140(3). pii: e20170670. doi: 10.1542/peds.2017-0670. Epub 2017 Aug 16. Physician-Nurse Interactions in Critical Care. Khowaja-Punjwani S, Smardo C, Hendricks MR, Lantos JD). Overall, nearly 20% of nurses leave their first job within a year of being hired (ref. Kovner CT, Brewer CS, Fatehi F, Jun J. What does nurse turnover rate mean and what is the rate? Policy Polit Nurs Pract. 2014;15(3–4):64–71).‍ Poor communication leads to medical errors (Aiken LH, Clarke SP, Sloane DM, Lake ET, Cheney T. Effects of hospital care environment on patient mortality and nurse outcomes. J Nurs Adm. 2008;38(5):223–229).*

Page 13; line 245: Being a HCP and researcher from one of the Scandinavian countries this is a bit hard to understand. Please shortly describe the differences in context in the methods-section under “Study Context and Participants”.

***Reply to reviewer’s #4 comment:***

Thank you for your valuable comment. Please find in the section under ”Study Context and Participants” short description:

“Due to different professional and academic workload and obligations of HCPs and other HCPs between secondary and tertiary level institutions we expect that different ethical dilemmas will be perceived within each groups and subgroups of HCPs and other HCPs.

Page 15; lines 264-265: Another reason for writing about other ways of dealing with ethical dilemmas then HECs. From previous research I have learnt that there is a great value of shared inter-professional ethics case reflections.

***Reply to reviewer’s #4 comment:***

*We agree with the reviewer’s comment.*

Page 16, Table3: Reaction? Please clarify what is intended.

Suggestion: Association between types of HCPs and ways of dealing with ethical dilemmas.

***Reply to reviewer’s #4 comment:***

*Thank you for your suggestion, we gladly accepted it and replaced it.*

Page 16, Table3: This comes for the second time and this time with different numbers? Please correct

***Reply to reviewer’s #4 comment:***

Correction was made. Corrected item: Contact the media

Page 18; line 284: What is a family circle? The patients family?

***Reply to reviewer’s #4 comment:***

*Family circle means HCPs’ family. Please find correction in the manuscript*

Page 19; Fig2: Please see above!

***Reply to reviewer’s #4 comment:***

*Correction was made. Corrected title is: Fig 2. Ways of dealing to ethical dilemmas…*

Page 19; lines 299: What are the standard procedures? If this should be written here it must be described in the background. Standard procedures could differ between countries, cities, hospitals, units etc.

***Reply to reviewer’s #4 comment:***

*Thank you for this comment. The question to the participants was whether the HCPs are aware of standard procedures for solving ethical dilemmas. Under hospital regulations’ acts, each Slovenian hospital should have a document how to resolve indivividual conflicts whether between HCPs or HCPs and patients. Moreover each hospital should also have a person who is dealing with such conflicts, i.e. ombudsman of patient rights, complaint board, as well as Hospital Ethics Committee. In the UMC Ljubljana safety officers for patients, employees, visitors and others are employed.*

*Whenever somebody perceives professional deviation, whether ethical or nonethical , an institution should have ombudsam, committee or safety officers to whom anybody can addreess complaints.*

Page 19; line 300: Yes of being unaware, or being aware? Please clarify!

***Reply to reviewer’s #4 comment:***

*The correct answer is: yes of being aware*

Page 19; Table 4: Aware or unaware of? (yes: aware; no: unaware)

***Reply to reviewer’s #4 comment:***

*Please find corrected version in the manuscript and table:*

*Yes of being aware*

*No of being unaware*

Page 20; line 320: Usually, sentences do not start with numbers. Please check writing guidelines.

***Reply to reviewer’s #4 comment:***

*Correction was made in the manuscript: More than one third (36.5%) of all…*

Page 23; Table 6: Please specify from were these roles were collected. Are there a common service or role description for the 14 hospitals? How is HECs practice performed

***Reply to reviewer’s #4 comment:***

*Thank you for your comment. Our* *predetermined ethical dilemmas chosen in the questionnaire were based on our previous student research research work in the biggest tertiary hospital in Slovenia in 2015. In this research we tested and validated what are the most important ethical dilemmas. Please see below a reference:*

*(ref. KUČAN, Rok, GROŠELJ, Jon. Delovanje področnih komisij za medicinsko etiko v zdravstvenih zavodih v Sloveniji : soočanje zdravstvenih delavcev z etičnimi dilemami in z delovanjem komisije za medicinsko etiko Univerzitetnega kliničnega centra Ljubljana. Ljubljana: [T. Šket], 2015. VI, 49 f., graf. prikazi, tabele. [COBISS.SI-ID* [*2800044*](https://plus.si.cobiss.net/opac7/bib/2800044?lang=si)*])*

DISCUSSION:

Page 25; lines 355-377: Physicians? A nurse can be a doctor to when earned a PhD.

***Reply to reviewer’s #4 comment:***

*We agree with the reviewer's comment. Please find changes in the entire manuscript.*

Please see above, other needs to be defined.

***Reply to reviewer’s #4 comment:***

*Corrections have been made in the manuscript with definitions of otherHCPs.*

This needs to be explained or removed, “the nature” is on a further philosophical level.

***Reply to reviewer’s #4 comment:***

*Correction has been made. “the nature” was removed.*

Please clarify, maybe misunderstanding due to language?

***Reply to reviewer’s #4 comment:***

*Please find corrected text: ...and what is the way of dealing resolving ethical dilemmas.* *This study reveals several important findings.*

This is really interesting, a lot of other studies confirm the opposite. What are the underlying interpretation of the phenomenon?

***Reply to reviewer’s #4 comment:***

*Thank you for your comment. We really don’t know the cause and the right answer.In postsocialistic health system in Slovenia physicians are under great pressure to serve all health expectations from patients “offered” them by health politics while limited resources are available. The lack of physicians and modern technology for diagnostics make physicians dissatisfied and under moral distress when they cannot fulfill the expectations of the patients.*

Correct English?

Comparable with the 20 predefined ethical dilemmas in the present study? Or just defined as ethical dilemmas and not specified.

***Reply to reviewer’s #4 comment:***

*This is correct statement.The “various” means defined as ethical dilemmas and not specified.*

Almost 15 year old reference.

***Reply to reviewer’s #4 comment:***

*We believe that this reference is still valid for the purpose of discussion.*

Page 26; line391-393: Which indeed is a great argument for educating physicians but also nurses and other HCPs

***Reply to reviewer’s #4 comment:***

*We can no more than to agree.*

How is this related to the results in the present study? Please problematize!

***Reply to reviewer’s #4 comment:***

*This results concur with ours where we found that 35.3% od physicians working in the tertiary level hospitals would discuss with hospital ethical committee compared to only 4% of them in secondary level hospitals when facing with ethical dilemma (p=0.015). No differences were found for nurses and other HCPs. Therefore we can draw a conclusion that not only physicians in the secondary level hopsitals but also nurses and other HCPs in secondary and teriary level hospitals are probably not enough aware of presence and therefore using hospital ethics committees when facing with ethical dilemmas. More education is probably needed as well spreading of information among HCPs in the hospitals that HECs are available and ready to help in solving ethical dilemmas.*

Page 26; lines400: Very nice discussion

***Reply to reviewer’s #4 comment:*** *for this comment.*

Page 27; line 417-418: What is the interpretation of this? Are they as close to the patient and the suffering as the physicians and nurses? Do they see the patient in another way?

***Reply to reviewer’s #4 comment:***

*The answer cannot be interpreted unanimously. What we can derive from this answer is that we should conduct a new research in which we would take questions on this issue raised. One of the possible answers would be a.) that the otherHCPs have access to large amount of data, information whent they take care of the patients specifically to their profession and that seems to them the most important, b.) that nobody teach them how to manage and interpret many personal information they came to their ears.*

Page 28; line448-449: ref?

***Reply to reviewer’s #4 comment:***

*No reference is available*

.Page 29; lines 466-467: Please clarify as the statement is hard to understand? What do you mean with “…are available only when considering the reasons for consultations with HECs” ?

***Reply to reviewer’s #4 comment:***

*Thank you for your comment. Please find corrected text: “Data information from Moeller et al study how HCPs solve ethical dilemmas after they reviewed 100 cases of……..”.*

Page 30; lines483-484: Repetition, please correct.

***Reply to reviewer’s #4 comment:***

*Correction made! Please find that we replaced "nurses" with "to those"*

Page 30; lines 502-504: Doesn´t this first say that none of the other HCPS consulted the HECs and then in the second sentence that 6.4% did it anyway?

***Reply to reviewer’s #4 comment:***

*Please find my correction. We added additional sentence for clarification: “Among the physicians working in the tertiary level institutions 15 (33,3%) consulted with HECs in 2014”.*

Page 31; lines 502-504: According to my opinion: Just because no one uses the services does not mean that it does not matter. The fundamental work of the HECs seems to need a review and efforts have to be made to inform the HCPs about the support that the HEC can offer.

***Reply to reviewer’s #4 comment:***

*We agree with the reviewer’s opinion and we removed this sentence.*

Page 31; lines 511-513: Isn´t this a circle argument? The most important role is in concordance with the most important role? Please clarify!

***Reply to reviewer’s #4 comment:***

*We agree with the reviewer’s opinion and we removed this sentence.*

LIMITATIONS:

Page 31; lines 523-527: However, do You believe that it is possible to explore the same research question in such different contexts`? The ethical dilemmas are varying leading to different needs of ethics support. According to literature ethics is not context dependent but still ethics support needs to be tailored based on the experiences perceived by the HCPs working in that specific care institution. Imagine the difference between hospitals for mental diseases and intensive care units in regards to for example the time limit the dilemma has to be resolved within. Enabling scientific stringency my perception is that support services should be studied in the same type of care and cultural affiliation.

***Reply to reviewer’s #4 comment:***

*Thank you fo your valubl comments. We removed thi sentence form the text.*

Page 32; lines 531-532: This could be future research

***Reply to reviewer’s #4 comment:***

*Agree with the reviewer's comment. We reoved this sentence and leave it for future research*

ACKNOWLEGEMENTSch!

Page 32; lines 546-547: This is results, what conclusion are You drawing out of these results? What does it mean?

***Reply to reviewer’s #4 comment:***

*Thank you for the reviewer’s comment.*

CONCLUSIONS:

Page 32; lines 550: As authors? This can be read as You only are thanking physicians that answered the questionnaire. I think it is polite to be thankful to all the participants for contributing with their experiences by completing the questionnaire. Consider adding.

***Reply to reviewer’s #4 comment:***

*We corrected acknowledgment. Please find it in the manuscript. “We thank all the Slovenian health-care providers (physicians, nurses and all others who take care for the benefit of the patients in the hospitals) for their kind participation in the study and the ~~Slovene Society of Intensive Medicine~~ management of hospitals for cooperation and support.*

*”*

ACKNOWLEDGEMENTS:

Page 32; lines 550-551:

As authors? This can be read as You only are thanking physicians that answered the questionnaire. I think it is polite to be thankful to all the participants for contributing with their experiences by completing the questionnaire. Consider adding.

***Reply to reviewer’s comment:*** *We agree with the reviewer’s comment, however this was type writing error and was corrected accordingly. Please find corrected sentence: “**We thank all the Slovenian health-care providers (physicians, nurses and all others who take care for the benefit of the patients in the hospitals) for their kind participation in the study and the ~~Slovene Society of Intensive Medicine~~ management of hospitals for cooperation and support.* “
